# Supplementary material for: The Innate Immune Sensor Zbp1 Mediates Central Nervous System Inflammation Induced by Angiostrongylus Cantonensis by Promoting Macrophage Inflammatory Phenotypes
Source: Adv Sci (Weinh). 2025 Jan 24;12(11):2413675. doi: 10.1002/advs.202413675 (PMC11923990; doi:10.1002/advs.202413675)
Supplement: Supplementary file 1 — Supporting Information [file ADVS-12-2413675-s001.docx]

Title Page

The Innate Immune Sensor Zbp1 Mediates Central Nervous System Inflammation Induced by Angiostrongylus Cantonensis by Promoting Macrophage Inflammatory Phenotypes

Hongli Zhou, Minyu Zhou, XiPing Liao, Liangyu Zhang, Hang Wei, Yuting Lu, Yiqing Zhang, Hui Huang, Yue Hu, Tao Chen*, Zhiyue Lv*

T. Chen, Z. Lv

Department of Neurology, Hainan General Hospital and Hainan Hospital of Hainan Medical University, Haikou, Hainan 570311, China.

Email: Zhiyue Lv: lvzhiyue@mail.sysu.edu.cn

Tao Chen: ctxwyc@163.com

H. Zhou, M. Zhou, Y. Lu, Y. Zhang, H. Huang, Y. Hu, Z. Lv

Key Laboratory of Tropical Disease Control (Sun Yat-Sen University), Ministry of Education, Guangzhou, Guangdong 510080, China.

H. Zhou, X. Liao

Clinical Medical Research Center, the Second Affiliated Hospital, Army Medical University, Chongqing, China.

M. Zhou, L. Zhang, Y. Lu, Y. Zhang, H. Huang, Y. Hu, Z. Lv

Provincial Engineering Technology Research Center for Biological Vector Control, Guangzhou 510080, China.

Z. Lv

Key Laboratory of Tropical Translational Medicine of Ministry of Education, Hainan Medical University, Haikou, Hainan 570216, China.

^*^Correspondence

Abstract

Angiostrongylus cantonensis (AC) is the leading cause of eosinophilic meningoencephalitis worldwide. The neuroimmune interactions between peripheral and central immune systems in angiostrongyliasis remain unclear. In this study, we observed significant infiltration of eosinophils, myeloid cells, macrophages, neutrophils, and Ly6C monocytes in the brains of AC-infected mice, with macrophages being the most abundant. RNA-seq and SMART-seq analysis of pattern recognition receptor (PRR) and DNA sensor gene sets revealed a marked increase in Z-DNA binding protein 1 (Zbp1) expression in infected mice. Confocal microscopy, RT-qPCR, western blotting, and immunohistochemistry further confirmed that Zbp1 was specifically upregulated in macrophages and microglia. Using Zbp1-knockout mice and flow cytometry, we found that knockout of Zbp1 enhanced lymphocyte infiltration and natural killer cell cytotoxicity, modulating the immune microenvironment in the CNS during AC infection. Mechanistically, we revealed that in macrophage Zbp1 directly binds to RIP3 to promote its phosphorylation, subsequently facilitating the phosphorylation of Mlkl protein. The activated Zbp1-pRIP3-pMlkl axis leads to necroptosis and upregulates pro-inflammatory cytokines including TNF-α, IL-1α, CXCL9, CXCL10 in macrophages, which recruits and activates immune cells. These findings offer new insights into the pathogenic mechanisms of angiostrongyliasis and suggest potential therapeutic strategies.

**Keywords:** AC; Zbp1; macrophages; polarization; immune microenvironment

1.Introduction

Neuroinflammation is a key component of neurological disorders, in which there are substantial interactions between immune responses and processes between cells of the central nervous system (CNS) and the periphery.^[1]^ In cases of neuroinflammation such as glioma, Alzheimer's disease, viral central nervous system infections, cerebral ischemia, and encephalitis, pathogenic immunity can disrupt the structure and function of the CNS.The crosstalk between the resident immune cells, including microglia and astrocytes, and infiltrating peripheral immune cells is a crucial factor.^[2–6]^ Peripheral immune cell entry into the CNS was tracked by cell tracing technology,^[7]^ and T-cell-deficient mice manifest cognitive deficits, demonstrating a key role of the immune cells in brain development and functions.^[8]^

Neuroinflammation caused by neuro-parasite infection is a specific type of neuroinflammation, which is still an important source of incidence rate and mortality worldwide. After cerebral infection with the parasite, such as Toxoplasma gondii, Plasmodium falciparum, Taenia solium, and Angiostrongylus cantonensis (AC), the worm exploits hormones, growth factors and other host-produced molecules to promote their own development and immune evasion.^[9]^ Various immune cells, including monocytes, eosinophils (Eos), macrophages, and neutrophils (Neu), subsequently infiltrate the brain of the helminth-infected host, initiating a primarily T helper 2 (Th2)-type immune response, which may protect the host from the detrimental inflammatory response in the brain.^[10]^ Natural killer (NK) cells can migrate to the CNS in the pathophysiological processes of cerebral ischemia, trauma, or infection, exerting direct cytotoxic effects on target cells or pathogens.^[11–13]^ Similarly, γδT cells can directly induce immune responses to pathogens and enhance the function of αβT cells by weakening the immunosuppressive effect of regulatory T cells (Tregs).^[14]^ AC specifically invade the CNS of the host and cause significant eosinophilic meningoencephalitis, accompanied by considerable infiltration of monocytes and NK cells in the brain and a significant secretion of interferon (IFN)-γ and tumor necrosis factor (TNF)-α, resulting in cytotoxic effects.^[15]^ Moreover, as a response to AC infection, γδT cells contribute to brain demyelination of the host by secreting interleukin (IL)-17A.^[16]^ However, the dynamic changes and functions of brain myeloid macrophages and their interactions with other immune cells, such as NK cells and T cells, are still not entirely clear.

It is becoming increasingly accepted that macrophages are crucial in tumor immune evasion, parasitic infection, viral infection, and neurodegenerative diseases.^[17]^ Because macrophages are highly heterogeneous and can differentiate in specific microenvironments, their functions are multifaceted. In early-stage lung cancer, alveolar macrophages accumulate near cancer cells to promote cancer cell invasion and induce increased Tregs to enhance tumor immune evasion,^[18]^ and M2 macrophage-derived exosomes promote cell migration and invasion in colon cancer.^[19]^ Macrophages can also induce gemcitabine resistance by releasing deoxycytidine, a compound with a structure similar to that of gemcitabine.^[20]^ In addition to secreting metabolites, macrophages themselves can regulate the host immune microenvironment through programmed cell death. Macrophages that undergo programmed cell death release signals to the microenvironment, thereby modulating the proportion of lymphocytes in the microenvironment and their cytotoxic effects.^[17]^ However, the profiling of cell death of brain macrophages in the host infected by AC and the role of the macrophages in regulating immune microenvironment in the brain remain unclear.

Z-DNA binding protein 1 (Zbp1), also known as DAI and DLM-1, is an important pathogen sensor that contains a Zα domain, a Zβ domain, a receptor-interacting protein (RIP) homotypic interaction motif, and a Z-DNA binding domain. Recent studies have identified Zbp1 as an innate sensor of infections and a target of pathogen evasion strategies in viral infections, parasitic infections, autoimmune diseases and inflammatory diseases.^[21–25]^ The molecule is activated by sensing host or viral nucleic acids through the nucleic acid domain to induce apoptosis or necrotic apoptosis in host cells, thereby inhibiting viral replication. Upon influenza A virus (IAV) infection, Zbp1 binds to IAV genomic RNA and localizes to specific locations within the cell,^[26]^and it is simultaneously ubiquitinated,^[27]^ thereby activating RIP3 and inducing RIP3 phosphorylation in the nucleus to induce cell death. On the other hand, phosphorylated RIP3 simultaneously activates mixed lineage kinase domain-like protein (Mlkl). Mlkl then triggers destruction of the nuclear membrane and facilitates the leakage of nuclear DNA into the cytoplasm and the transport of activated Mlkl to the cell membrane to mediate necrotic apoptosis of the cells.^[28]^ Zbp1 inhibits inflammation in Toxoplasma gondii infection, and Zbp1-knockout (KO) macrophages secrete higher levels of proinflammatory cytokines than wild-type macrophages in response to T. gondii infection. Furthermore, Zbp1-knockout mice exhibit a greater cyst burden and stronger inflammatory cytokine response during chronic infection compared to control mice.^[29]^ Our previous study demonstrated that obvious apoptosis or necrotic apoptosis occur in the CNS of the hosts infected with AC,^[30]^ however, physiological role of the ability of Zbp1 in the neuroparasitic infection remains to be elucidated.

In this study, we first investigated the dynamic changes in innate immune cells in the brain after AC infection in non-permissive hosts. The pattern recognition receptor (PRR) and DNA sensor gene sets involved in the antipathogen response were analyzed using transcriptome data from AC-infected mice. Zbp1 levels in brain neurons, astrocytes, oligodendrocytes, and microglia/macrophages were comprehensively investigated using laser confocal microscopy, and immunofluorescence colocalization analysis. Expression of Zbp1 in AC-infected hosts was determined by qPCR, western blot, and immunohistochemistry. Finally, using Zbp1-knockout mice as a model, flow cytometry was applied to assess immune functions of brain macrophages and characteristics of the immune microenvironment of brain during the infection. Combining the above methods with primary cell induction experiments, the role of Zbp1 in regulating macrophage-lymphocytes in the microenvironment was further elucidated to better profile the host brain inflammatory immune microenvironment after AC infection and to provide a scientific basis for the optimization of treatment strategies for angiostrongyliasis.

2.Results

2.1. Dynamics of Brain Mononuclear Cells and Granulocytes in Mice Infected with AC

To investigate the dynamic changes in brain mononuclear cells and granulocytes after AC infection in unsuitable hosts, we infected mice with third-stage AC larvae for 7, 14, and 21 days, and mouse brains were collected for assessment of Eos, myeloid cell, macrophage, Neu, Ly6C^low^ monocyte (Ly6C^low^), and Ly6C^hi^ monocyte (Ly6C^hi^) levels using flow cytometry. **Figure 1A** shows the gating strategy for flow cytometry. F4/80^+^ SiglecF^+^ were Eos, F4/80^+^CD11b^+^ were macrophages, and CD11b^+^CD45^+^ were myeloid cells. Neu were labeled with CD11b^+^CD45^+^F4/80^-^Ly6G^+^, Ly6C^low^ monocytes were labeled with CD11b^+^CD45^+^F4/80^-^Ly6G^-^Ly6C^low^, and Ly6C^hi^ were labeled with CD11b^+^CD45^+^F4/80^-^Ly6G^-^Ly6C^hi^. The results revealed no Eos in the brains of uninfected mice, as their proportion was close to 0. The percentage of Eos increased to 0.038% and 0.118% in mice 7 and 14 days after AC infection, respectively. By 21 days after infection, brain Eos levels had increased sharply to 3.805% (P < 0.0001, Figure 1B). Consistent with the alteration in Eos, the proportion of myeloid cells in the brains of normal mice (the 0-day group) was close to 0. The proportion of myeloid cells in the brains of mice gradually increased at 7 days and 14 days after infection with AC, peaking (5.11%) 21 days after infection (P < 0.0001, Figure 1C). Myeloid-derived cells include macrophages, Neu, and Ly6C monocytes. Among them, the proportion of macrophages was highest on day 21 after infection, at 3.67% (P < 0.0001, Figure 1D). The proportion of Neu in the brain gradually ascended with infection time as well, reaching 0.3% 21 days after infection (P < 0.0001, Figure 1E). The percentages of Ly6C^hi^ and Ly6C^low^ monocyte infiltration gradually elevated with infection progression and peaked 21 days after infection, at 0.058% (P < 0.0001) and 0.52% (P < 0.0001, Figure 1F,G), respectively. The results indicate that the brain immune microenvironment clearly changes after AC infection. There is a small amount of infiltration of myeloid cells, mononuclear cells, and granulocytes in the early stage of infection, and the infiltration increases sharply 21 days after infection, which participates in the complex immune response of the host brain.

2.2. Zbp1 is Specifically Highly Expressed in the Brain Macrophages of AC-infected Mice

The above results indicated that after AC infection, increased bone marrow-derived myeloid mononuclear cells and granulocytes migrated to the brain to exert immunomodulatory functions. Among them, macrophages, as antigen-presenting cells, not only function as natural immune cells but also regulate the adaptive immune process through antigen presentation.^[31]^ After AC infection, many bone marrow-derived macrophages (BMDMs) enter the brain, but their specific functions remain unclear. In the innate immune response, macrophages can recognize pathogens, microbial-specific pathogen-associated molecular patterns (PAMPs) or damage-associated molecular patterns (DAMPs) through PRRs, thereby inducing innate immune responses. Common PRRs are primarily divided into the following categories: Toll-like receptor (TLR), RIG-1 receptor (RLR), NOD-like receptor (NLR) and C-type lectin receptor (CLR).^[32,33]^ DNA sensor–related genes are also important in the response and regulation of pathogen infection.^[34]^ We used the gene expression microarray data (accession number: GSE159486) to analyze different PRRs and DNA sensor gene sets. The results demonstrated that the TLR, RLR, DNA sensor, NLR, and CLR genes were upregulated to varying degrees (Figure 1H,S1A-D). The most significantly upregulated genes were Clec4e, Clec7a, and Zbp1. Clec4e and Clec7a are CLR involved in a variety of parasitic infections.^[35–37]^ As a key DNA sensor, Zbp1 has rarely been reported in parasitic infections and its function and mechanism is still poorly understood in parasitic diseases.^[38]^ Therefore, this study primarily focused on Zbp1. Zbp1 was significantly upregulated by approximately 120-fold after AC infection (P < 0.05, Figure 1I), suggesting that it may have an important regulatory function in the pathological damage of AC-infected mice.

As a nucleic acid sensor, Zbp1 is widely involved in the occurrence and progression of pathogen infection, autoimmune diseases, and inflammatory diseases.^[24–28]^ Zbp1 plays a key antiviral role in macrophages,^[29]^ but whether it mediates the function of macrophages after AC infection is unknown. First, it was necessary to determine the cell types with high Zbp1 expression in response to AC infection. To this end, we performed immunofluorescence colocalization experiments on mouse brain sections 21 days after AC infection. The results indicated that after AC infection, Zbp1 (green fluorescence) was not significantly colocalized with the molecular marker of oligodendrocytes (myelin basic protein, MBP) (**Figure 2A**). We used NeuN as a marker of neurons, one of the most important cell types in the brain, to perform colocalization analysis with Zbp1. The results revealed no significant colocalization of red and green fluorescence (Figure 2B). Astrocytes, one of the most abundant cell types in the brain, are important in the occurrence and progression of disease.^[39]^ We selected GFAP as a marker for astrocytes to investigate the colocalization of astrocytes with Zbp1 and found that astrocytes did not express Zbp1 (Figure 2C). Finally, we employed Iba1 as a marker for microglia/macrophages, and the fluorescence colocalization results demonstrated significant colocalization between Iba1-positive cells and Zbp1-positive cells (yellow, Figure 2D). Further colocalization analysis using ImageJ software indicated that the fluorescence colocalization coefficient of oligodendrocytes, neurons, astrocytes, and Zbp1 was only 0.2, while the coexpression index of microglia/macrophages and Zbp1 was 0.8 (Figure 2E,F). These results indicate that after AC infection, mouse brain microglia/macrophages specifically express Zbp1.

To further examine the expression of Zbp1 in microglia/macrophages after AC infection, we performed in vitro experiments using BMDMs and the microglial cell line N9. Because Zbp1 belongs to the interferon gene family, IFN-γ was selected as the IFN-specific positive control, while LPS was used as the nonspecific positive control. As shown in **Figure 3A,B**, IFN-γ induced high expression of Zbp1 in microglia and BMDMs. Zbp1 upregulation was observed 4 hours post-infection, with BMDMs exhibiting higher sensitivity to IFN-γcompared to microglia (Figure 3A,B). However, after LPS stimulation, Zbp1 levels in microglia and BMDMs were approximately 10- to 25-fold higher (Figure 3C,D), indicating that the effect of LPS on Zbp1 expression in the two cell lines was very similar, without specificity. After the cells were stimulated with larvae soluble antigen (LSA), the levels of Zbp1 in microglia were twofold, threefold, and twofold higher at 4, 8, and 12 h, respectively, after stimulation and decreased to normal levels 24 h after stimulation (Figure 3E). The levels of Zbp1 in BMDMs 4, 8, 12, and 24 h after stimulation were fivefold (P < 0.0001), 17-fold (P < 0.0001), 17-fold (P < 0.001), and eightfold (P < 0.05) higher, respectively (Figure 3F), indicating that the Zbp1 level of BMDMs was higher and that the response was stronger than that of microglia after LSA stimulation which may be determined by their gene expression characteristics. These results suggest that Zbp1 is primarily expressed in BMDMs in the mouse brain after AC infection.

2.3. Dynamic Changes in Zbp1 Occur in the Brains of Mice Infected with AC

To further explore the changes in Zbp1 in the mouse brain after AC infection, we first assessed levels of Zbp1 using western blotting and found that protein levels of Zbp1 were approximately 10-fold higher 21 days after AC infection (**Figure 4A**, P < 0.0001). RT‒qPCR further confirmed this result (Figure 4B). At the same time, immunofluorescence confirmed that after AC infection, the number of Zbp1-positive cells significantly increased (Figure 4C). To further investigate the dynamic changes in Zbp1 levels after AC infection, we also performed RT‒qPCR and western blot experiments on the brain tissues of mice 0, 7, 14, and 21 days after infection with AC. To further investigate the dynamic changes in Zbp1 levels after AC infection, we also performed RT‒qPCR and western blot experiments on the brain tissues of mice 0, 7, 14, and 21 days after infection with AC. The levels of Zbp1 mRNA and protein did not change significantly on day 7 after infection, but significantly upregulated on both the 14 and 21 days after infection (Figure 4D,E), The apparent inconsistency between the trends in panels D and E on days 14 and 21 may be due to post-translational modifications of Zbp1 or the post-transcriptional regulation at 21 days post infection. Immunohistochemistry confirmed that the number of Zbp1-positive cells significantly increased after AC infection (Figure 4F,G). This upregulation was positively correlated with the activation and infiltration of microglia and macrophages in the brain after AC infection. In response to viral infection, high expression of Zbp1 is closely related to RIP3 activation, which may trigger the apoptosis and necroptosis of cell. As preciously report, phosphorylated RIP3 can simultaneously activate Mlkl. Transport of activated Mlkl to the cell membrane mediates the occurrence of necrotic apoptosis.^[25,26]^ Consistent with previously report，the results of endogenous co-IP experiments on mouse brain tissues after AC infection showed significant enrichment of the Zbp1 protein in mouse brain tissues, while the IP-alone samples exhibited no GAPDH bands (Figure 4H). As shown, there was a significant enhancement in the binding of Zbp1 to RIP3 (Figure 4H). Studies have shown that Zbp1 can promote necroptosis in macrophages^[29,40]^. Consistently, our results indicated that stimulation with LSA led to a significant increase in the expression of RIP3, pRIP3, and pMlkl. However, upon the knockout of Zbp1, these markers of necroptotic cell death were markedly reduced, suggesting that Zbp1 can promote necroptosis in BMDMs following LSA stimulation via RIP3/Mlkl pathway. Our data suggest that Zbp1 may be involved in the programmed cell death of microglia/macrophages after AC infection.

2.4. Zbp1 Promotes Necroptosis of Macrophages in Mouse Brain

We further investigated the function of Zbp1 in microglia/macrophages after AC infection. We constructed Zbp1-knockout mice and verified the knockout efficiency of Zbp1 by western blot. The results showed that after AC infection, the protein expression of Zbp1 significantly increased, while the levels were notably reduced in both the KONC (the uninfected AC group of Zbp1-knockout mice) and KOAC groups (the infected AC group of Zbp1-knockout mice) (**Figure S2**). Next, wild type (WT) mice and Zbp1-knockout mice were infected with AC, mice were sacrificed on day 21, and macrophages in the brains of the WTAC (the infected AC group of wild-type mice) and KOAC groups were sorted and subjected to SMART-Seq sequencing. The results demonstrated that compared to the WTAC group, the KOAC group exhibited 156 significantly upregulated genes and 92 significantly downregulated genes in response to Zbp1 knockout (**Figure 5A**). The GSEA results for differentially expressed genes indicated that genes involved in the cell cycle, cell death, and antigen presentation were significantly enriched (Figure 5B,C), confirming earlier results (Figure 4H) and suggesting that Zbp1 knockout reduces cell death of macrophages; furthermore, the gene set involved in the inhibition of cell death was downregulated (Figure 5D), and the antigen presentation ability of macrophages was weakened.

Next, we carried out CD45 and CD11b staining to identify microglia (CD45^int^ CD11b^int^). F4/80 and CD11b were used to label macrophages, and the proportions of necrotic and apoptotic cells were determined using Annexin V and PI staining. The results revealed a slight increase of necrotic cells in microglia after infection (0.05%, P < 0.05), but there was no significant difference in the KOAC, while microglial apoptosis was significantly increased after AC infection (1%, P < 0.0001). However, after Zbp1 knockout, the percentage of apoptotic microglia decreased significantly (P < 0.0001) (Figure 5E,G). Macrophage staining revealed that in the WTNC (the uninfected AC group of wild-type mice) and KONC, there were fewer macrophages in the brain, and the proportion of cell death was close to 0. In contrast, after AC infection, the percentage of apoptotic macrophages in mouse brain increased from 0% to 31% (P < 0.0001), while a significant decrease (15%, P < 0.001) in apoptotic macrophages was detected in infected Zbp1 knockout mice (Figure 5F,H). More importantly, after AC infection, the proportion of macrophage necrosis significantly accumulated to approximately 0.9% (P < 0.0001). However, in Zbp1-knockout mice, the proportion of necrotic cells significantly decreased from 0.9% to 0.01% (P < 0.001) (Figure 5F,H), demonstrating Zbp1-dependent necroptosis of macrophages in brain of the host infected by AC.

2.5. Zbp1 Promotes Lymphocytic Infiltration in the Mouse Brain

Macrophages is critical in regulating the immune microenvironment, mediating immune cell interactions and pathogen clearance.^[17,22,23]^ To understand the effect of Zbp1 on the immune microenvironment of the host brain after AC infection, we first assessed dynamics of Eos, Neu, myeloid cells, macrophages, and Ly6C monocytes in the brains of mice after AC infection. The results were consistent with previous findings, the infiltration of Eos, Neu, myeloid cells, macrophages, and Ly6C monocytes in the brains of WT mice without Zbp1 knockout significantly increased. However, compared with WTAC, the infiltration of various cells in KOAC mice infected with AC did not show significant differences(**Figure S3A-F**). This indicates that the increased infiltration of these innate immune cells is not related to Zbp1. Therefore, We subsequently examined changes in antigen-presenting cells and lymphocytes. CD11c^+^CD11b was used to label dendritic cells (DCs), NK1.1 was used to label NK cells, and CD3^+^CD4 and CD3^+^CD8 were used to label T cells (**Figure 6A**). The results revealed that the proportion of DCs increased from 0 to 0.45% (P < 0.0001) after AC infection and decreased to 0.35% (P < 0.001) in Zbp1-knockout brains (Figure 6B). The proportion of NK cells was upregulated to 1.4% after AC infection (P < 0.0001) but decreased to 0.45% in Zbp1-knockout mice (P < 0.0001) (Figure 6C). For T lymphocytes, the percentage of CD4 T cells and the percentage of CD8 T cells were upregulated to 0.6% (P < 0.0001) and 0.25% (P < 0.0001), respectively, after AC infection (Figure 6D,E) and were significantly reduced to 0.15% (P < 0.0001) and 0.14% (P < 0.0001) in Zbp1-knockout mice (Figure 6D,E). Peripheral blood flow cytometry results similarly showed that compared with WTAC, DCs in the blood of the KOAC group were significantly reduced (**Figure S4A**). Although NK and CD3^+^CD4 and CD3^+^CD8 T cells did not show significant differences between these two groups, NK and CD3^+^CD4 and CD3^+^CD8 T cells were significantly reduced in the knockout group compared to the non knockout group (Figure S4B-D). Taken together, these results indicate that Zbp1 promotes the infiltration of brain DCs and lymphocytes after AC infection and participates in the regulation of changes that occur in the brain immune microenvironment.

2.6. The Cytotoxicity of Brain Lymphocytes is Reduced after Zbp1 Knockout

Effects of Zbp1 on lymphocytes were assessed by measuring the levels of TNF-α and IFN-γ released by NK cells and CD3^+^CD4 and CD3^+^CD8 T cells (**Figure 7A**). The results demonstrated that NK cells and CD3^+^CD4 and CD3^+^CD8 T cells in the brains of mice secreted high levels of TNF-α and IFN-γ in response to AC infection (Figure 7B-G) to participate in the antipathogen response. No obvious changes were detected in TNF-α and IFN-γ productions by CD8 T cells in Zbp1 knockout mouse model (Figure 7B,C). The levels of TNF-α secreted by CD4 T cells declined, while the expression of IFN-γ did not change significantly (Figure 7D,E). In contrast, levels of TNF-α secreted by NK cells decreased from 1.6% to 0.7% (P < 0.0001), and levels of IFN-γ decreased from 0.41% to 0.3% (P < 0.0001, Figure 7F,G), suggesting that Zbp1 deficiency primarily attenuates the cytotoxicity of NK cells.

2.7. Zbp1-knockout Macrophages Regulate Lymphocyte Infiltration via Chemokines and Inhibit NK-cell Cytotoxicity through M2 Polarization

To understand regulatory roles of macrophage Zbp1 in lymphocytes (CD4 and CD8 T cells) and NK cells, we presented in vitro coculture model of macrophage and T cells. As shown in **Figure 8A,** TNF-α and TNF-α+LSA were used to induce BMDMs in WT and Zbp1 KO groups. Since the spleen is rich in lymphocytes, the activated macrophages were colocalized with primary spleen cells. The levels of TNF-α and IFN-γ secreted by NK cells, CD3**^+^**CD4 and CD3^+^CD8 T cells were then assessed by flow cytometry. The results revealed that WT and KO macrophages were cocultured with splenocytes and stimulated by LSA, TNF-α and IFN-γ releases by CD4 and CD8 T cells was significantly enhanced, but there was no significant difference between the KO and WT groups (Figure 8B), indicating that the Zbp1 deficiency in macrophages does not affect the functional status of CD3^+^CD4 or CD3^+^CD8 T cells. In contrast, after LSA stimulation, TNF-α and IFN-γ secretion by NK cells were increased from 35% and 33% to 50% and 43%, respectively (P < 0.05, Figure 8C), while in macrophages, deletion of Zbp1 caused the levels of TNF-α and IFN-γ secreted by NK cells to decrease to 35% and 30% (P < 0.05, Figure 8C), indicating that loss of Zbp1 in macrophages inhibits the cytotoxicity of NK cells.

To elucidate the specific mechanism by which macrophage Zbp1 affects CD4 and CD8 T cells and regulates the cytotoxicity of NK cells, mRNA sequencing assay were performed. The results demonstrated that after Zbp1 knockout, the expression of macrophage chemokine-related genes was clearly altered (Figure 8D), and the levels of chemokines (Cxcl10, Ccl3, Ccl4, Cxcl10, Ccl4, Cxcl10, Ccl4, Cxcl10, Ccl4, Ccl5, and Ccl8) that recruit CD4 and CD8 T cells were significantly reduced, while expression of chemokines (Cx3cl1, Ccl6, Ccl7, Ccl9, and Cxcl12) that recruit macrophages, Neu, and myeloid-derived suppressor cells was significantly increased (Figure 8E), indicating that macrophages lacking Zbp1 inhibit the infiltration of CD4 and CD8 T cells by downregulating chemokines. In addition, the macrophage antigen-presenting gene was significantly downregulated (Figure 8F), suggesting that after Zbp1 deletion, macrophage antigen-presenting ability is obviously decreased. M1 macrophages participate in the promotion of NK cell cytotoxicity,^[35]^ while M2 macrophages inhibit NK cell viability in the microenvironment.^[36–38]^ Our results demonstrate that M1 macrophage–related genes are downregulated, while M2 macrophage-related genes are upregulated (Figure 8G).Moreover, the transcriptome data indicates that the level of TNF-α, a key cytokine in the NF-κB signaling pathway, was most notably decreased after the knockout of Zbp1 (Figure 8G). The NF-κB signaling pathway in macrophages plays an important role in their polarization process.^[41–43]^ We further examined the changes in the NF-κB signaling pathway. The results showed that LSA stimulation markedly activated the NF-κB pathway in macrophages, but Zbp1 deletion led to a notable decrease in pathway activity and cytokine levels (Figure 8H). This indicates that Zbp1 promoted macrophage polarization via the NF-κB pathway, aligning with recent studies.^[44,45]^ Collectively, these results indicating that Zbp1 can promote the M1 polarization of macrophages, thereby promoting the cytotoxicity of NK cells in the immune microenvironment.

3.Discussion

Neurological disorders, including meningitis, glioma, and neurodegenerative lesions are frequently linked to brain inflammation.^[46–48]^ Immune cells mediate the development of the CNS^[49]^ and are closely related to the progression of CNS diseases.^[50]^ There are multiple heterogeneous immune cells in glioma, with myeloid cells being the most important group of cells in the immune microenvironment of glioma.^[51]^ Myeloid-derived suppressor cells play a predominant role in the immune microenvironment and immune suppression of gliomas.^[51]^ BMDMs promote glioma progression by altering metabolism in the local microenvironment.^[52]^ And previous studies in patients with gliomas have shown an increase in Neu and Eos infiltration at the tumour site.^[53,54]^ Moreover, marked eosinophilic and neutrophilic infiltration is a common feature of parasitic infections.^[55]^ For example, Neu promote the inflammatory pathogenesis of malaria through neutrophil extracellular traps (NETs).^[56]^ Ascaris lumbricoides infection is characterized by pulmonary infiltrates with eosinophilia and mononuclear macrophage mediated Th2-type immune response.^[57]^ Similarly, the present study showed that noticeable Eos, Neu, and monocytes/macrophages infiltrated the brain, peaking 21 days after AC infection, consistent with previous results.^[58]^ In addition, macrophages, the most abundant myeloid cells, have various functions in development,^[59]^ tissue repair and regeneration,^[60]^ and are key components of the immune response to infectious organisms.^[61]^ Macrophages can promote the inflammatory response to infections and participate in tissue repair through polarization.^[62]^ In response to AC infection, many macrophages infiltrate the brain, where they participate in antiparasitic immunity.

Zbp1, also known as DAI, is a type of DNA sensor that can be activated after sensing host or viral nucleic acids to induce apoptosis or necrosis in host cells.^[63]^ It is widely involved in the progression of infections and inflammatory diseases.^[64]^ Zbp1 stimulates pulmonary Neu infiltration in the hosts infected with influenza virus.^[65]^ After T. gondii infection, deletion of Zbp1 induces macrophages to secrete increased proinflammatory cytokines and reduce NO synthesis, which in turn leads to an increase in the burden of cysts in Zbp1-knockout mice during chronic T. gondii infection.^[66]^ Similar to these results, we found that Zbp1 levels in the brain increased sharply 14 and 21 days after AC infection. Immunofluorescence colocalization results and in vitro stimulation experiments confirmed that Zbp1 was specifically expressed in only macrophages and was involved in the regulation of macrophage function. IFN-γ and LPS stimulation of peritoneal macrophages significantly induce the expression of Zbp1,^[28]^ and knockdown of Zbp1 reduces LPS-induced lung pathological damage by reducing the levels of inflammatory cytokines.^[67]^ Specifically, we observed that microglia and BMDMs exhibit differential sensitivity in Zbp1 expression in response to LSA stimulation. Studies reveal that in brain infections caused by Trypanosoma brucei, BMDMs and microglia have distinct responses to inflammation, likely due to differences in immune receptor expression.^[68]^ BMDMs' higher plasticity may facilitate the formation of functionally diverse macrophage populations.^[68]^ In Trichomonas brucei infections, microglia and BAM boost blood-brain barrier permeability and monocyte recruitment, but microglia limit macrophage dispersion in acute demyelination.^[69]^ This underscores the importance of the disease context in macrophage interactions and the need for further research into BMDM interactions with tissue-resident macrophages across diseases. Zbp1 is also involved in LPS/Z-VAD-induced necrotic apoptosis of macrophages.^[28]^ In this study, our immunoprecipitation assay demonstrated a strong binding of Zbp1 to RIP3, the necroptotic marker. After Zbp1 was knocked out, the proportion of necroptotic macrophages was significantly reduced, suggesting that Zbp1 promotes necroptosis of macrophages in brain of the infected host. This study found that in a degenerative disease model induced by neuroinflammation resulting from AC infection, Zbp1 is highly expressed in macrophages and exerts a pro-inflammatory effect through the RIP3-Mlkl axis, Consist with our conclusion, many studies reported that Zbp1 mediated necroptosis via pRIP3-pMlkl,^[70-72]^ the Mlkl signaling and necroptosis led to the increase of pro-inflammatory cytokines in macrophage.^[73,74]^ Additionally, we noted that related studies have highlighted the potential role of Zbp1 in other neurodegenerative diseases. For example, in a neuroinflammation model of Parkinson's disease, Zbp1 plays an anti-inflammatory role in glial cells and may be involved in regulating cell death.^[75]^ In contrast, in Alzheimer's disease, Zbp1 is upregulated in neurons, promoting neuronal damage, pyroptosis, oxidative stress, and inflammation.^[76]^ These findings suggest that the role of Zbp1 in different neurodegenerative diseases shares certain similarities but also presents differences, indicating that Zbp1's function is context-dependent, relying on specific disease environments and cell types. Therefore, future therapeutic strategies targeting Zbp1 in neurodegenerative diseases should carefully consider these potential differences.

Increasing studies have demonstrated that necroptosis in macrophages plays a significant role in modulating the immune microenvironment. For example, Macrophages regulate immune responses by phagocytosing apoptotic cells or inducing their own programmed cell death.^[77,78]^ Apoptosis and polarization of alveolar macrophages promote lung injury in septic rats,^[79]^ and necroptotic macrophages can induce severe inflammatory responses by secreting inflammatory cytokines.^[80]^ Necroptosis of macrophages promotes the occurrence and progression of rheumatoid arthritis, and inhibition of the A20 protein can protect the body against rheumatoid arthritis by inhibiting macrophage death.^[81]^ Upon AC infection, we observed a marked increase in brain macrophage death along with heightened infiltration of T cells, NK cells, and DCs. However, macrophage cell death significantly dropped following Zbp1 knockout, in line with published studies that Zbp1 knockout significantly reduces host cell apoptosis and necrosis in HSV, MVCV, and IAV infection.^[28,65,66]^

After macrophages undergo cell death, the functions of nearby immune cells can be regulated through the release of antibacterial proteins.^[82]^ In our study, the transcriptomic analysis revealed that the expression of chemokines such as Cxcl10 that recruit CD4, CD8, NK, and DCs was significantly reduced in Zbp1-knockout macrophages, Concurrently, Zbp1-knockout macrophages exhibited a weakened M1 phenotype and an enhanced M2 phenotype, suggesting that Zbp1 regulates the infiltration of NK cells and T cells into the immune microenvironment by regulating macrophage polarization and chemokine releasing. Similarly, in tuberculosis, macrophages that undergo programmed cell death promote the cytotoxicity of T cells in the immune microenvironment through the release of apoptosis-related substances, resulting in reduced secretion of IFN-γ and TNF-α by T cells.^[83]^ Zbp1-deficient macrophages significantly reduce the levels of TNF-α and IFN-γ secreted by NK cells under LSA stimulation, which was related to the polarization state of the macrophages. M1 macrophages participate in the promotion of NK cell cytotoxicity,^[84]^ while M2 macrophages inhibit NK cell viability in the microenvironment,^[85–87]^ suggesting that macrophages can regulate the tissue immune microenvironment through changes in their polarization state.

In this study, we investigated the dynamic changes in brain immune cell levels at different times after AC infection and found high levels of granulocyte, monocyte, and macrophage infiltration into the brain. Transcriptome analysis and laser confocal microscopy colocalization experiments revealed that Zbp1 mRNA and protein were specifically highly expressed in macrophages in the brains of infected mice. Knockout of Zbp1 reduced macrophage necrotic apoptosis and increased the expression of chemokines and M2-type polarization, thereby reducing the infiltration of CD3^+^CD4 T cells and CD3^+^CD8 T cells in the brain and reducing the cytotoxicity of NK cells, alleviating the acute inflammatory response (**Figure 9**). However, there are still some limitations. This study is primarily focused on AC, which may not fully capture the range of host responses to various pathogens. This underscores the need for a more extensive validation. Future research should aim to expand the investigation of Zbp1 across a broader spectrum of neuroinflammatory models to better understand its role in macrophage biology. Including human samples in these studies would enhance their relevance to real-world disease conditions, offering a more precise reflection of clinical scenarios.

We propose for the first time that Zbp1 participates in the immune response to helminth infection. Zbp1 modulates lymphocyte infiltration and NK cell cytotoxicity by regulating the necroptosis, cytokine secretion, and M1 polarization of macrophages in AC infection. The above phenotype is mediated by the binding of Zbp1 to RIP3 to promote its phosphorylation, and then facilitate Mlkl phosphorylation to activate Zbp1-pRIP3-pMlkl axis. The findings provide new insights into the crosstalk between innate immunity and adaptive immunity, deepen the understanding of the regulation of brain inflammation in angiostrongyliasis, and attempt to supply novel therapeutic strategies for treating the disease.

4.Experimental Section

Parasites and animal infections: Biomphalaria glabrata containing third-stage AC larvaewere maintained in our laboratory. The shell of B. glabrata was crushed using forceps, and the snail muscle tissue was fully removed using scissors. The digestion solution (2 g pepsin, 7 ml concentrated hydrochloric acid, 1000 ml distilled water) was added at 4 ml/g of tissue, and the mixture was placed in a 37 °C incubator for 30 min. Digestion was terminated by adding two volumes of normal saline, and third-stage larvae were counted under a stereomicroscope.

Female C57/BL6 mice (6-8 weeks of age, 18-20 g)(All animals were housed in a specific pathogen–free environment. The protocols were approved by the Laboratory Animal Welfare and Ethics Committee of Sun Yat-sen University.) were purchased from Beijing Charles River, and Zbp1-knockout mice were generated by the Experimental Animal Center of East China University of Science and Technology. Each mouse was infected with 30 AC larvae by gavage and sacrificed at the corresponding time point, and samples were collected for subsequent experiments. All animals were housed in a specific pathogen–free environment. All procedures were approved by the Laboratory Animal Welfare and Ethics Committee of Sun Yat-sen University.

Preparation of LSA:Under sterile conditions, AC from the brains of mice infected for 21 days were collected and placed in sterile phosphate-buffered saline (PBS). After three washes, the parasites were homogenized on ice to release the soluble antigens. After centrifugation at 12,000 rpm, the supernatant was passed through a 0.22-μm sterile filter (Millipore, MA, USA). The antigen concentration was quantified using a bicinchoninic acid (BCA) assay kit (Beyotime, Wuhan, China).

Flow cytometry analysis of immune cells in brain: Mouse brains were separated and thoroughly homogenized and then filtered through a 70-μm cell strainer. After centrifugation at 500 g for 5 min, and the supernatant was discarded and the single-cell suspension was resuspended in PBS. Lineage markers (Siglec-F, F4/80, CD45, CD11b, Ly6C) (BioLegend, CA, United States) were used for myeloid cell surface phenotyping. CD3, CD4, CD8, CD11c and NK1.1 (BioLegend) were used to detect the infiltration of NK cells, DCs, and T cells into the mouse brain. To determine cell death of brain macrophages, cells were stained with antibody cocktail (CD45, CD11b, F4/80 and Annexin V) (BioLegend) at 4 °C for 40 min, followed by centrifugation at 500 g for 5 min. Cell pellets obtained were resuspended in 500 μl of 1× binding buffer (BioLegend) after three washes, finally incubated with propidium iodide (PI) dye (BioLegend). To measure the levels of TNF-α and IFN-γ secreted by T cells and NK cells in the brain, cells were first stained with the mixture of antibodies (CD3, CD4, CD8, and NK1.1) (BioLegend), after fixation and centrifugation, the cell pellets obtained were further incubated with anti- mouse TNF-α and anti-mouse IFN-γ (BioLegend) for 40 min. Cell sorting and analysis were performed using CytoFLEX (Beckman Coulter, Atlanta, USA). Data analysis was performed using FlowJo software (Tree Star, San Carlos, USA).

Immunofluorescence:The brain tissues were fixed in 4% paraformaldehyde for 24 h and embedded in paraffin, then sectioned serially. The sections were subjected to antigen retrieval using ethylenediaminetetraacetic acid (EDTA, PH=8.0), then treated with 0.1% Triton X-100 for 15 min prior to blocking in 3% bovine serum albumin (BSA) at room temperature for 1 h. The corresponding primary antibodies including anti-rabbit NeuN (1:200, Cell Signaling Technology, Danvers, MA, United States), anti-mouse Zbp1 (1:50, Santa Cruz, CA, United States), anti-rabbit Iba1 (1:200, Abcam, Cambridge, UK), anti-rabbit GFAP (1:200, Cell Signaling Technology) and anti-rabbit MBP (1:200, Cell Signaling Technology) were incubated overnight at 4 °C respectively. Next, secondary antibodies anti-rabbit Alexa Fluor® 594 Conjugate (1:500, Cell Signaling Technology) and anti-mouse Alexa Fluor® 594 Conjugate (1:500, Cell Signaling Technology) were incubated at room temperature for 1 h. The sections were coverslipped with mounting medium containing DAPI. Subsequently photographed under LSM880 confocal laser-scanning inverted microscope (ZEISS, Jena, German). The fluorescence intensity was measured with the use of ImageJ and ZEN software (v2.3 lite, Carl Zeiss).

Coimmunoprecipitation (Co-IP):For co-IP assays, fresh mouse brain tissues were treated with IP standard lysis buffer (Thermo Fisher Scientific) supplemented with a mixture of protease and phosphatase inhibitors (1:1000, Thermo Fisher Scientific), and then incubated at 4 °C for 30 min. After centrifugation at 12,000 rpm for 15 min, supernatants were quantified with using the BCA protein concentration assay kit (Beyotime), then 400 μg of protein were incubated with 2 μg of antibody at 4 °C for 4 hours. Thereafter, 40 μl of protein G agarose beads (Roche, Basel, Switzerland) were incubated at 4 °C overnight. After washing 5 times with pre-chilled PBS containing protease and phosphatase inhibitor, the proteins were denatured using 1% sodium dodecyl sulfate (SDS), and the supernatant was then used for immunoblotting analysis.

RT-qPCR:Total RNA from mouse brain tissues or cell lines was extracted using TRIzol reagent (Invitrogen, Carlsbad, CA), the concentration and purity were determined in using a NanoDrop One (Thermo Fisher Scientific). cDNA was synthesized according to the manufacturer’s protocols (Thermo Fisher Scientific). The rReal-time quantitative PCR (qPCR) reaction was performed as follows: 95 °C, 10 min; and 40 cycles of 95 °C and 60 °C, 1 min. Quantitative detection was performed using SYBR Green (TaKaRa, Dalian, China) in the a LightCycler 480 Real-Time PCR system (Roche Diagnostics, Reinach, Switzerland). The primers used in this study are listed in Table 1. The gene expression levels were calculated by the 2^-ΔΔCT^ method and normalized to β-actin as described previously. ^[88]^

Western blot:Mouse brain tissues were lysed for 5 min in RIPA buffer (Thermo Fisher Scientific) containing protease and phosphatase inhibitors (1:1000, Thermo Fisher Scientific). The protein concentration in the solution was measured using a BCA reagent kit (Beyotime). 20μg of total protein were electrophoresed and fractionated by SDS–polyacrylamide gel, then transferred to a polyvinylidene fluoride (0.22 μm) membrane (Merck Millipore, MA, USA), and blocked in 5% non-fat milk in Tris-buffered saline with Tween-20 (TBST) at room temperature for 2 h. After blocking, membranes were incubated with the corresponding primary antibodies at 4 ℃ overnight and then incubated with the secondary antibody at room temperature for 1 h. The bands could be visualized with enhanced chemiluminescence detection kit (ECL) (Merck Millipore) using the chemiluminescence imaging analysis system (Tanon, Shanghai, China) according to the manufacturer's instructions. gapdh was chosen as the loading control.

Immunohistochemistry (IHC):Mouse brain were dissected and fixed in 4% paraformaldehyde, then embedded in paraffin and sectioned. The brain sections were acquired according to the methods in our previous study.^[30]^ For immunohistochemistry, sections were blocked with 3% bovine serum albumin (BSA) at room temperature for 1 h prior to incubation with Zbp1 mAb (AdipoGen, Seoul, Korea) overnight at 4 °C, then incubated with an HRP-conjugated secondary antibody (Cell Signaling Technology) at room temperature for 30 min. Subsequently, sections were stained with 3, 3′-diaminobenzidine (DAB) and aematoxylin. The stained sections were imaged under a light microscope (Leica, Heidelberg, Germany).

Induction of BMDMs:The bone marrow cells were extracted from femurs and tibia of C57BL/6 mice by gentle crushing in PBS. Cell pellets were obtained after filtration through a 70-μm cell strainer and centrifugation at 500g for 5 min. Cells were cultured in DMEM high-sugar glucose medium containing 10% FBS, 100 U/ml penicillin/streptomycin and 20 ng/μl macrophage colony-stimulating factor (MCSF) in a cell culture incubator at 37 °C with 5 % CO2. Half of the medium was changed every 2 days, and macrophages were induced on the day 7.

Coculture of BMDMs and splenocytes:The cell concentration of splenocytes was adjusted to 6×10^5^ cells/ml, and the cells were cultured in RPMI 1640 medium with 10% FBS and 100 U/ml penicillin/streptomycin. TNF-α (10 ng/ml) or TNF-α (10 ng/ml) + LSA (30 μg/ml) was used to stimulate BMDMs for 24 h; 3×10^5^ splenocytes were added to each well of a 12-well plate for coculture for 48-72 h. To determine the cytotoxic effects of T cells and NK cells, suspended splenocytes from the coculture system were collected for flow cytometry.

RNA extraction and RNA-seq:The inducted macrophages were collected for mRNA sequencing. Total RNA was extracted using magnetic beads and checked for a RIN number to inspect RNA integrity by an Agilent 2100 Bioanalyzer (Santa Clara, CA, US). Qualified total RNA was further purified by RNAClean XP Kit (Beckman Coulter, Inc. Kraemer Boulevard Brea, CA, USA) and RNase-Free DNase Set (QIAGEN, GmBH, Germany). VAHTS Universal V6 RNA-seq Library Prep Kit for Illumina® (Vazyme, Jiangsu, China) and VAHTS® mRNA Capure Beads (Mouse) (Vazyme) were used for generation and purification of cDNA library. RNA-Seq was performed on the Illumina NovaSeq6000 platform in paired-end configuration by Biotechnology Corporation (Shanghai, China).

Microglial cell culture: The mouse microglial cell line N9 was used for experiments in vitro. The N9 cell line was cultured in DMEM F-12 medium containing 10% FBS and 100 U/ml penicillin/streptomycin. The cells were cultured in an incubator at 37 °C and 5% CO2.

SMART-Seq and bioinformatics analysis: Single-cell suspensions were collected as described above. Total RNA was extracted from F4/80^+^CD11b^+^ cells sorted by high-speed flow cytometry system (MoFlo Astrios EQs). The SMART-Seq v4 Ultra Low Input RNA kit (Clontech Laboratories, Inc., USA) was used to synthesize cDNA following the manufacturer’s instructions. The final cDNA library was constructed by Illumina NovaseqTM 6000 sequence platform after purification. The sequence quality was verified using FastQC (http://www.bioinformatics.babraham.ac.uk/projects/fastqc/,0.11.9), including selection criteria Q20, Q30 and GC-content of the clean data. All raw sequence data have been deposited in NCBI Short Read Archive (SRA) with accession number PRJNA890398.

For bioinformatics analysis, the differentially expressed genes was screened by DESeq2 package between two different groups (and by edgeR between two samples). The genes with the parameter of false discovery rate (FDR) ＜ 0.05 and absolute fold change ≥ 2 were considered differentially expressed gene. Gene set enrichment analysis was performed using software GSEA (v4.1.0).

Statistical analysis: All data were expressed as mean ± standard deviation (SD). Statistical analyses were conducted using GraphPad Prism 7.00 (GraphPad Software, San Diego, CA, USA). Prior to any statistical tests, the distribution of each dataset was examined using the Shapiro–Wilk test to confirm normality. For comparisons between two independent groups, a two-tailed Student’s t-test was employed when the data satisfied assumptions of normality and homogeneity of variances. For comparisons involving more than two groups, a one-way analysis of variance (ANOVA) was performed, followed by Tukey’s post hoc test to address multiple comparisons and control the Type I error rate. A p-value below 0.05 was considered statistically significant. All tests were conducted in a two-tailed manner with an alpha level of 0.05.

Supporting Information

Supporting Information is available from the Wiley Online Library or from the author.

Acknowledgements

We thank Mr. Li Xiaobo and Mrs. Guan Yuanjun for valuable assistance of flow cytometry and confocal microscopy. This work was supported by grants from the National Natural Science Foundation of China (Grant No. 82072303), the Natural Science Foundation of Guangdong Province (No. 2024A1515011438), the Natural Science Foundation of Chongqing (Grant No. CSTB2023NSCQ-MSX0171).

Conﬂict of Interest

The authors declare no conﬂict of interest.

Availability of data and material

The data that support the findings of this study are available from the corresponding author upon reasonable request. All raw sequencing data have been deposited in the public database NCBI-Sequence Read Archive (SRA) under the BioProject accession number PRJNA890398 and PRJNA895686.


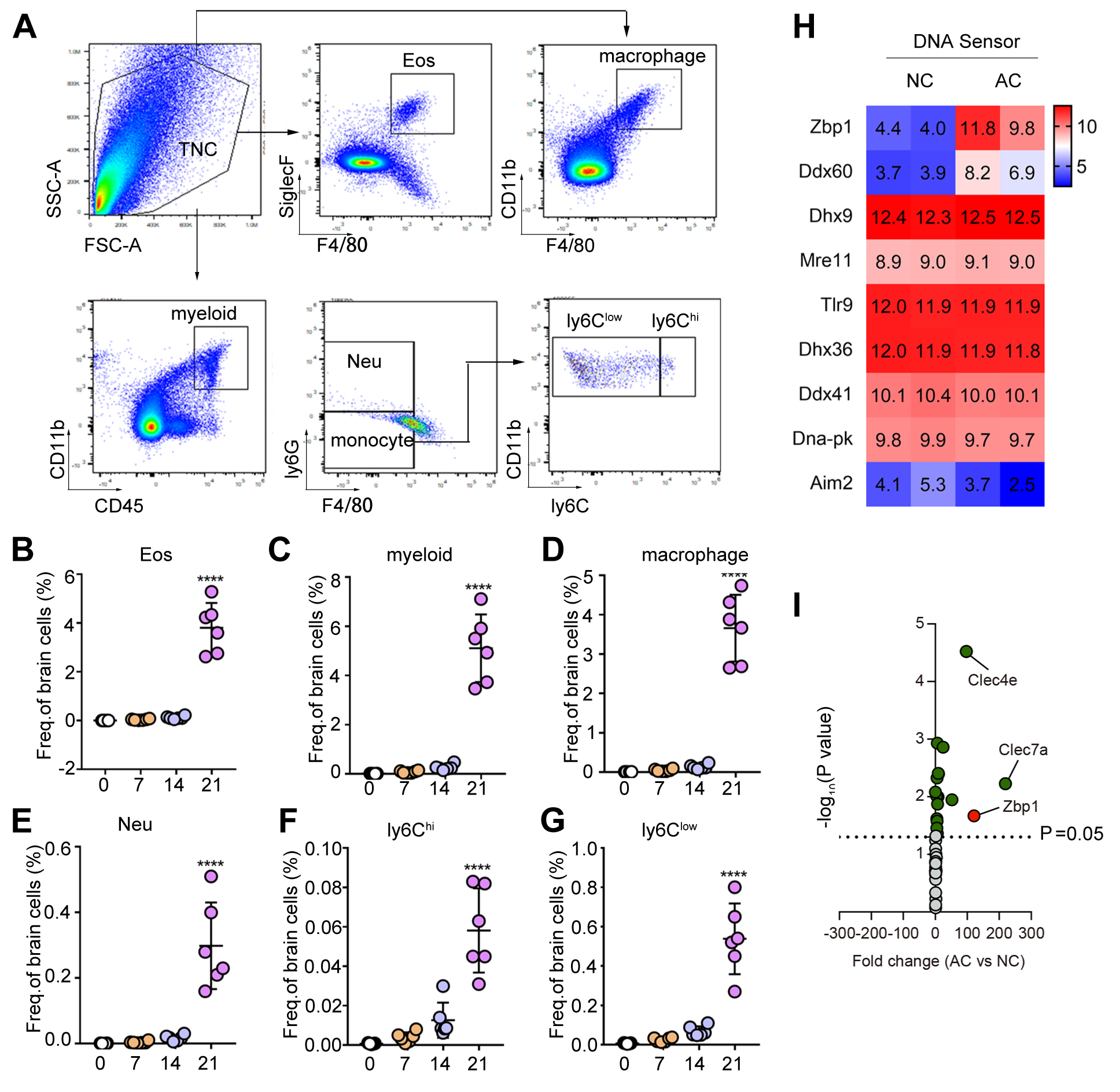


**Figure 1.** The brains of C57BL/6 mice exhibited dynamic changes in immune cells and altered expression profiles of DNA sensors after AC infection. (A) The granule cells (Eos and Neu) and monocytes (macrophage and ly6C monocytes) positive population was selected following FSC, SSC gating and single cell gating. (B-G) Proportion of Eos (B), myeloid cells (C), macrophage (D), Neu (E), ly6C^hi^ (F) and ly6C^low^ (G) monocytes at various time points (0, 7, 14, 21 days post infection) in mouse brain after AC infection as measured by FACS analysis. Eos: eosinophils. Neu: neutrophils. (H) The expression profiles of DNA Sensor in the brain of infected mouse displayed by heatmap analysis. (I) Differentially expressed genes between two groups shown in volcano plots. n=5-6 per group. ****, P < 0.0001, compare to 0 dpi.


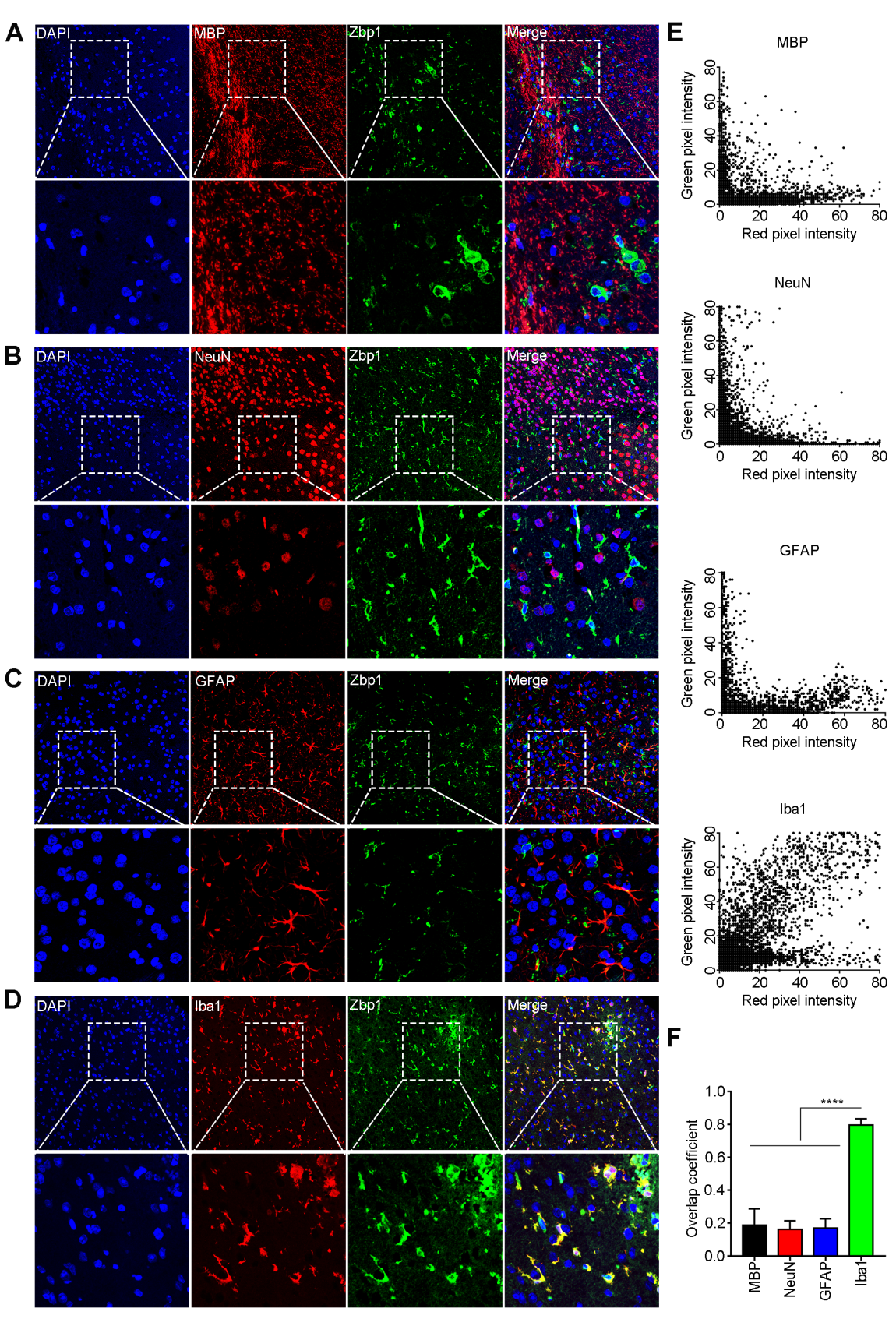


**Figure 2.** Zbp1 was specifically expressed in the brain macroglia/macrophages of AC-infected mice. (A-D) Immunofluorescence co-staining of Zbp1 and oligodendrocytes (MBP) (A), neurons (NeuN) (B), astrocytes (GFAP) (C), microglia/macrophage (Iba1) (D) to detect colocalization. (E) Co-localization analysis of red fluorescence (cell marker) and green fluorescence (Zbp1) in A-D using image J software. Each dot represents a pixel. The abscissa of the dot represents red fluorescence intensity, and the ordinate represents green fluorescence intensity. (F) The result showed the colocalization coefficient in E. **** P < 0.0001.


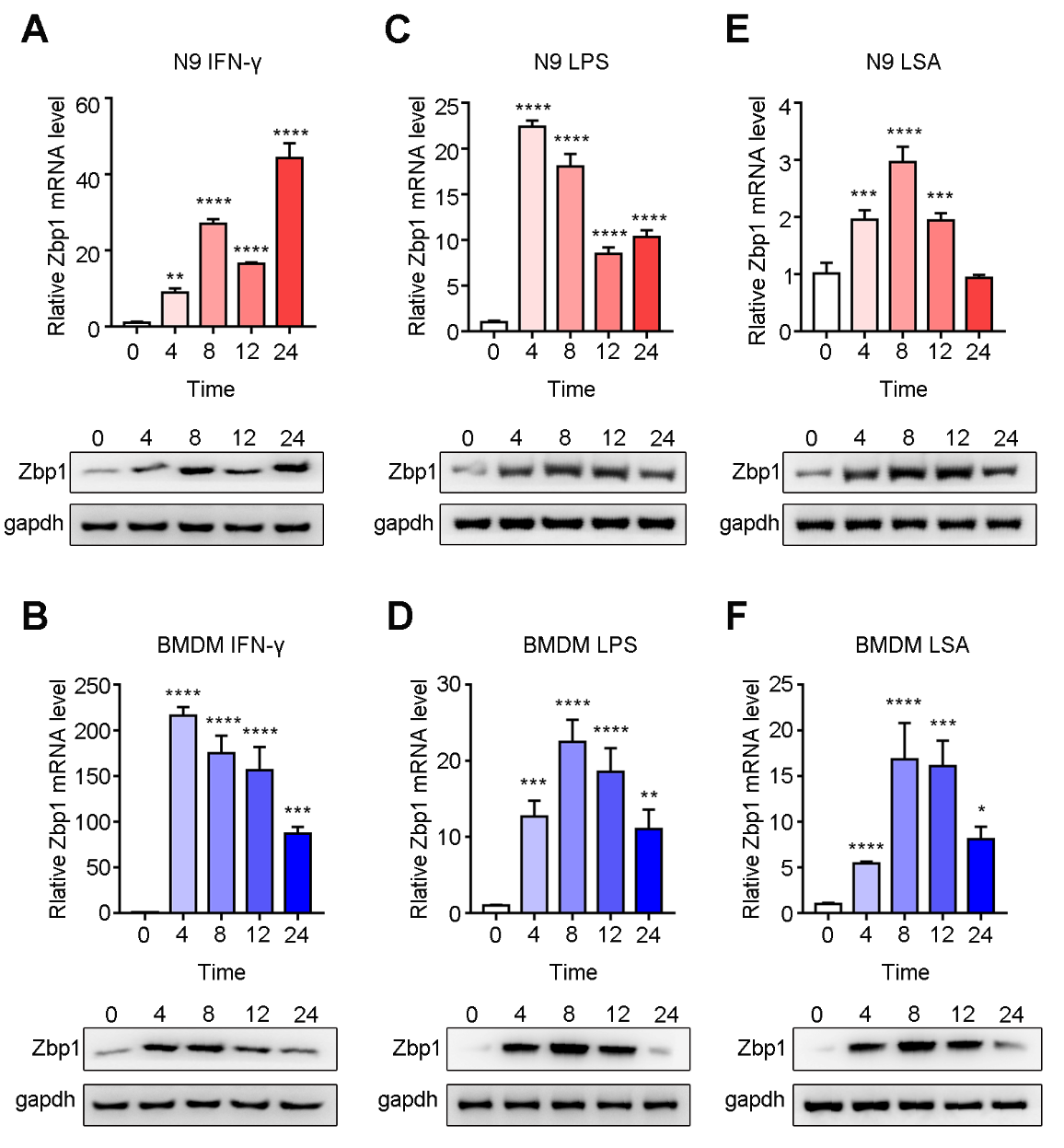


**Figure 3.** LSA induced high expression of Zbp1 in microglia and BMDM cells. The relative mRNA and protein levels of Zbp1 of microglia (N9) and bone marrow-derived macrophages (BMDM) stimulated with IFN-γ(A-B)、LPS(C-D) and LSA(E-F) at 0, 4, 8, 12, 24 hours. n=3 per group. *，P < 0.05，**，P < 0.01，***，P < 0.001，****，P < 0.0001, compare to 0 h.


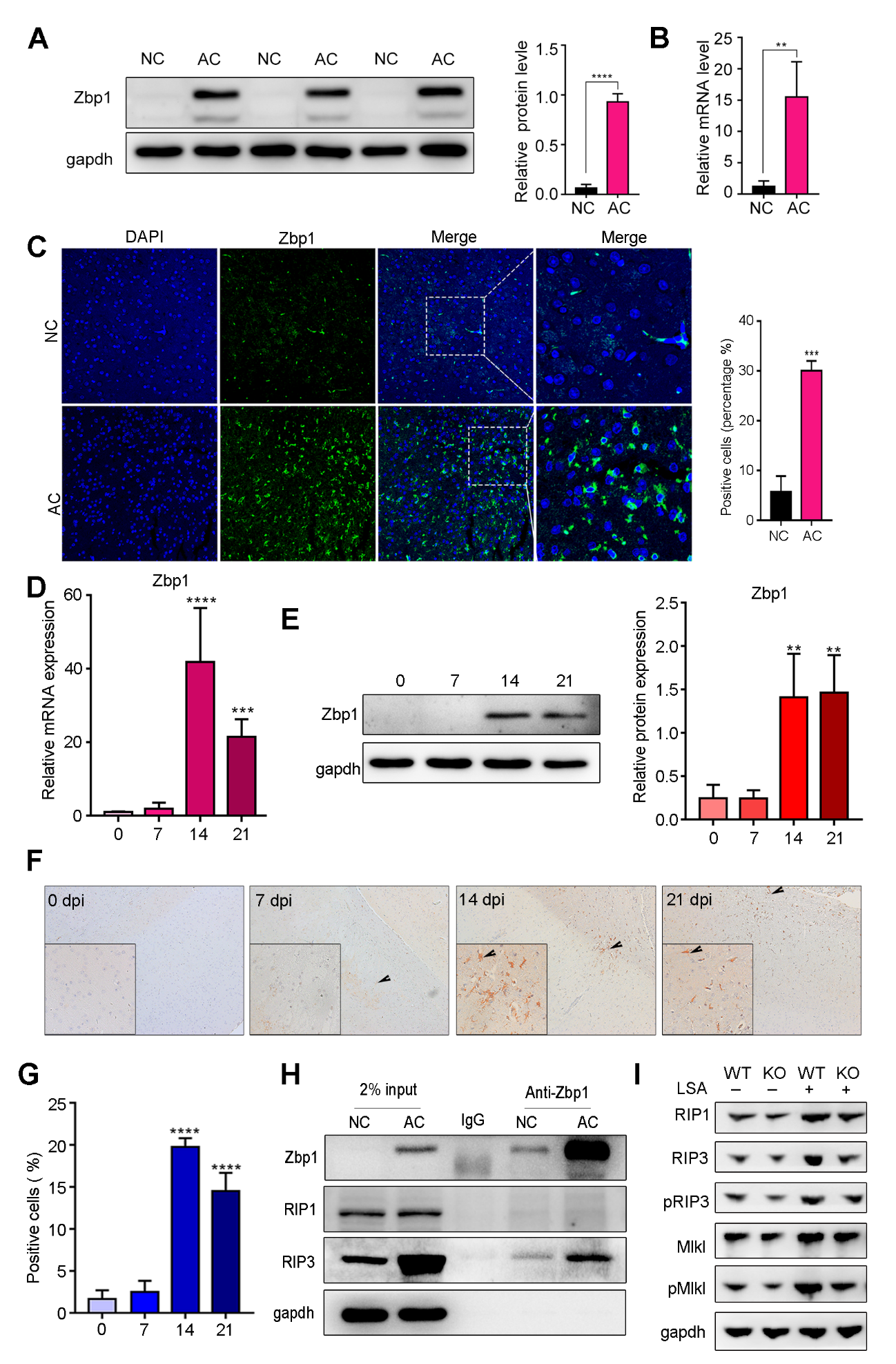


**Figure 4.** The relative expression of Zbp1 was elevated in mouse brain after AC infection. (A-B) the protein (A) and transcriptional (B) level of Zbp1 in mouse brain at 21 dpi was determined by western blot and RT-qPCR (n=5-6 per group). (C) The protein expression of Zbp1 were further assessed by immunofluorescence. (D-E) The translational (D) and transcriptional (E) level of Zbp1 in mouse brain at 0, 7, 14, 21 dpi (n=5-6 per group). (F-G) the localization and expression level of mouse brain at 0, 7, 14, 21 dpi were determined by IHC. (H) Co-immunoprecipitation of Zbp1, RIP1 and RIP3 by anti-Zbp1 antibody. (I) The protein expression of RIP1, RIP3, pRIP3, Mlkl and pMlkl. ** P < 0.01，*** P < 0.001，**** P < 0.0001 compare to 0 dpi。


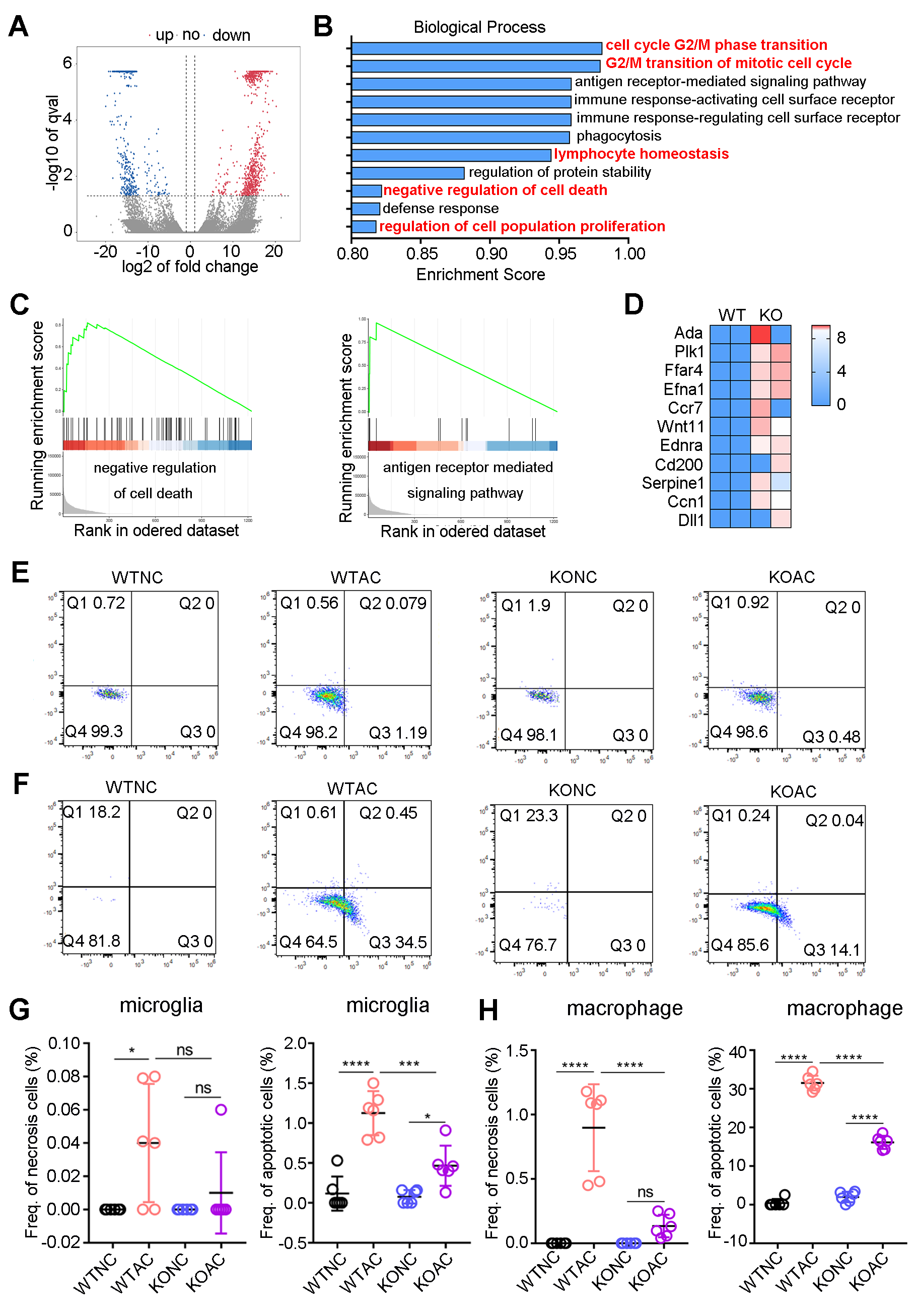


**Figure 5.** Zbp1 promoted the programmed cell death of microglia/macrophage in mouse brain after AC infection. (A) Differential expressed genes between WTAC (the infected AC group of wild-type mice) and KOAC (the infected AC group of Zbp1-knockout mice) shown in volcano plots. (B) Functional enrichment of the differential expressed genes. (C) Apoptotic pathway and antigen receptor mediated signaling pathway revealed by GSEA enrichment analysis. (D) Heatmap of genes involved in the negative regulation of cell death. (E, G) Representative image (E) and quantitative statistics (G) of apoptosis and necroptosis detection of microglia in mouse brain with Annexin V and PI in the different groups. (F, H) Apoptotic rate of macrophage in mouse brain was determined by flow cytometry, representative plots(F) and quantifications (H) were shown. n=5-6 per group. * P < 0.05，*** P < 0.001，**** P < 0.0001.


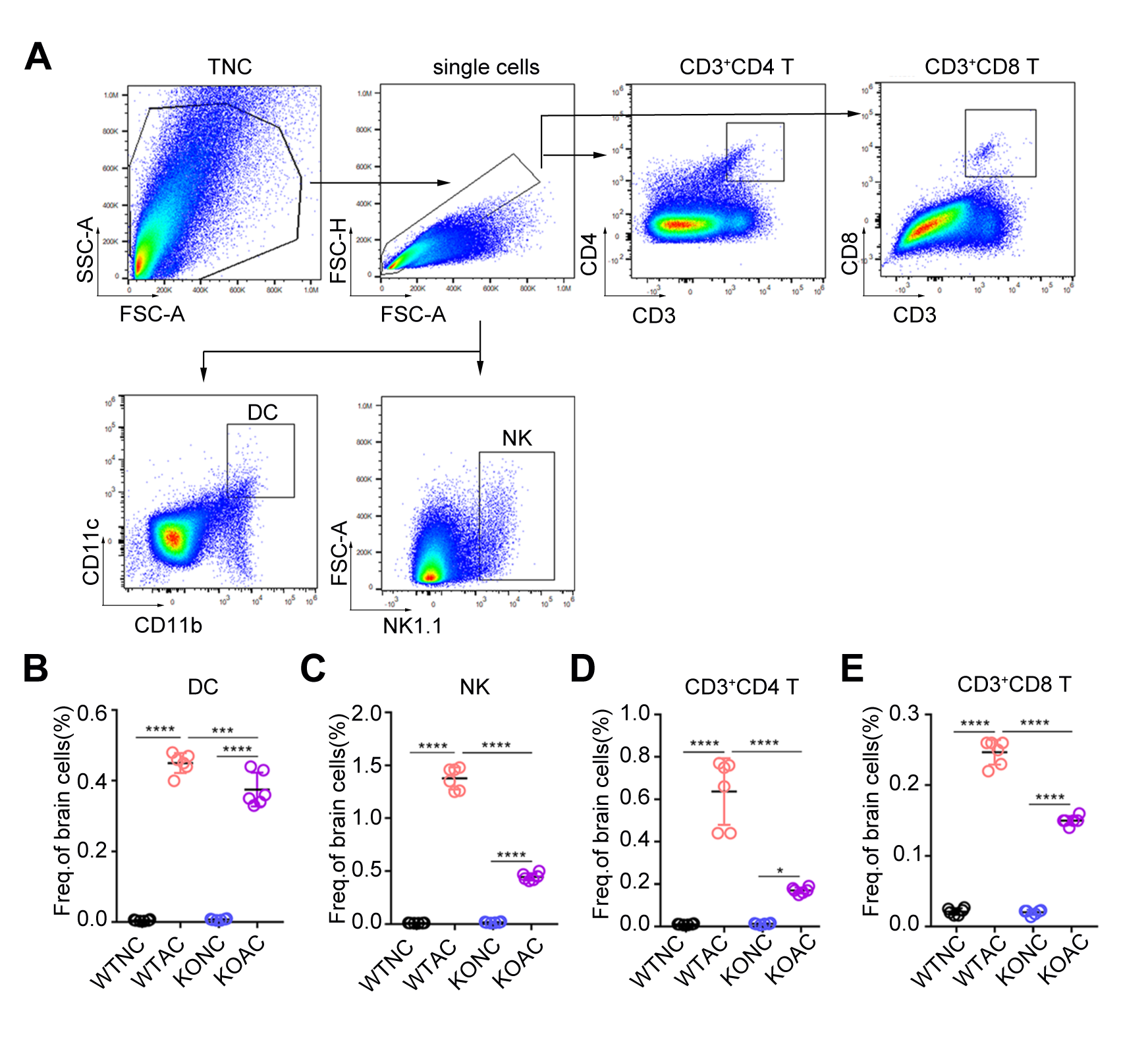


**Figure 6.** Zbp1-deficient mouse with AC infection exhibited reduced lymphocyte infiltration in brain. (A) Gating strategy of dendritic cell (DC), nature killer (NK) cells , CD3^+^CD4 and CD3^+^CD8 T cells of mouse brain. (B-E) The proportion of DC (B), NK(C), CD3^+^CD4(D) and CD3^+^CD8 T (E) cells in mouse brain from different groups. n=5-6 per group. * P < 0.05，*** P < 0.001，**** P < 0.0001.


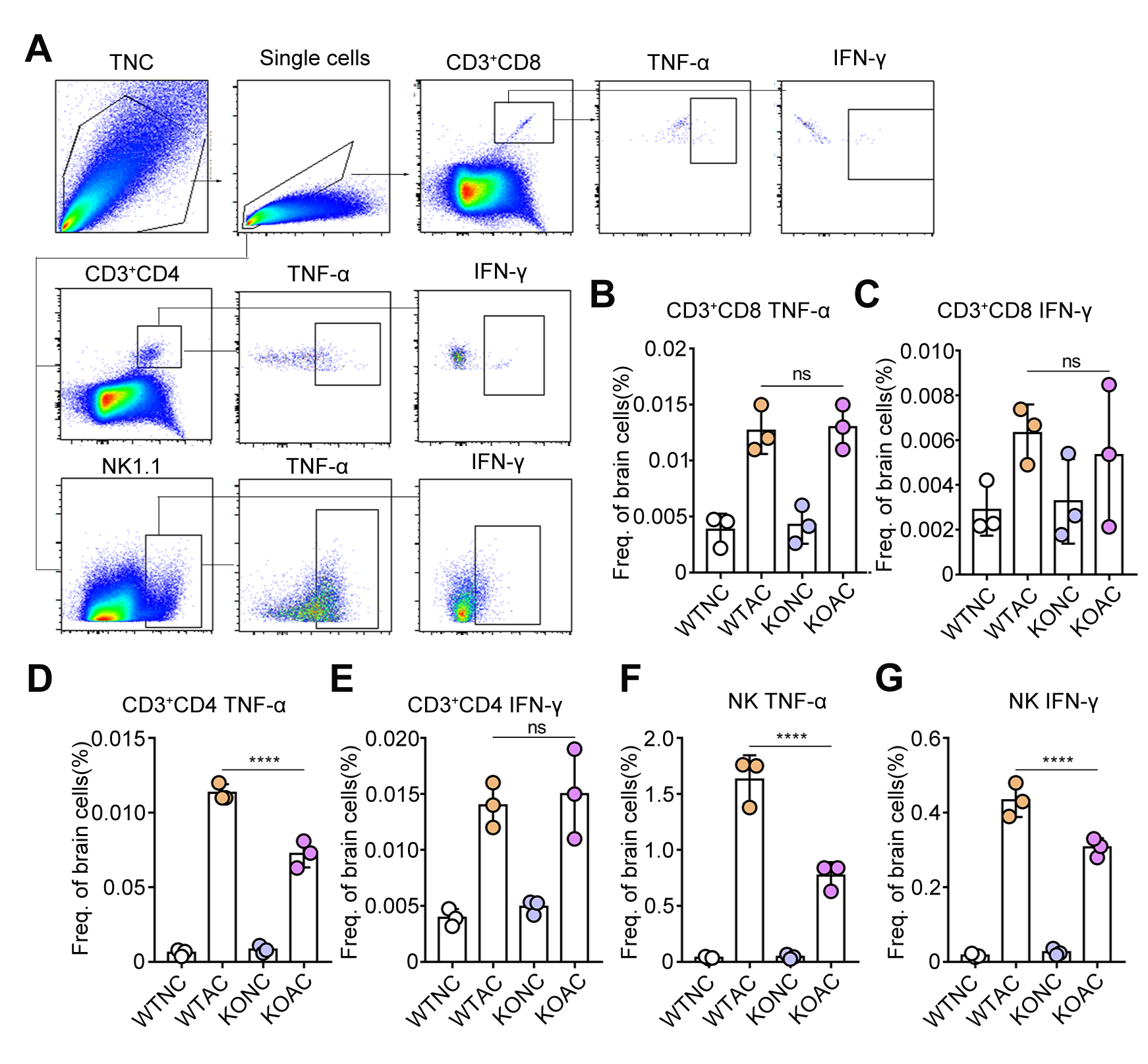


**Figure 7.** Zbp1-deficient mouse with AC infection exhibited reduced cytotoxicity of NK cells in brain. (A) Gating strategy showing the level of TNF-α and IFN-γ in NK, CD3^+^CD4 and CD3^+^CD8 T cells of mouse brain. (B-G) The level of TNF-α and IFN-γ in CD3^+^CD8 T (B-C) cells, CD3^+^CD4 T cells(D-E) and NK(F-G) in mouse brain from different groups. n=3 per group. **** P < 0.0001.


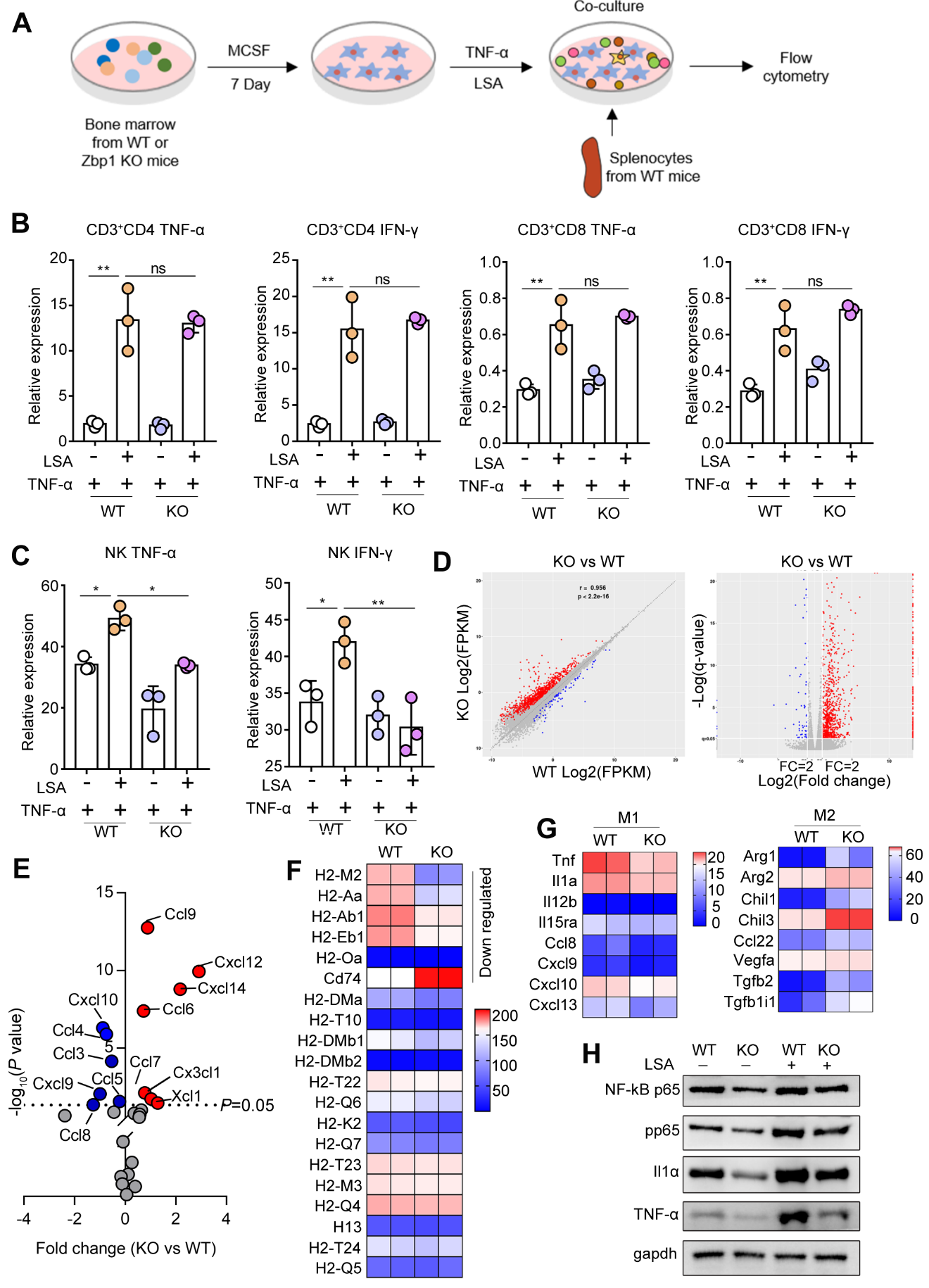


**Figure 8.** Infiltration of lymphocytes is regulated by chemokines and cytotoxicity of NK cells is inhibited by promoting the M2 polarization of Zbp1-deficient macrophage. (A) Diagrammatic sketch of co-culture experimental design. (B) The expression level of TNF-α and IFN-γ in CD4 and CD8T cells in different groups. (C) The expression level of TNF-α and IFN-γ in NK cells in different groups. (D) Volcano plots and scatter plots displaying genes detected by RNA-seq. (E) Expression of chemokines in macrophages with Zbp1 knock-out. (F) Expression of antigen presenting genes in macrophage from WT and KO group. (G) Heatmap of M1 and M2 polarization related genes in macrophage. (H)The translational levels of NF-kB p65, pp65, Il1αand TNF-α. n=3 per group. * P < 0.05, ** P < 0.01.


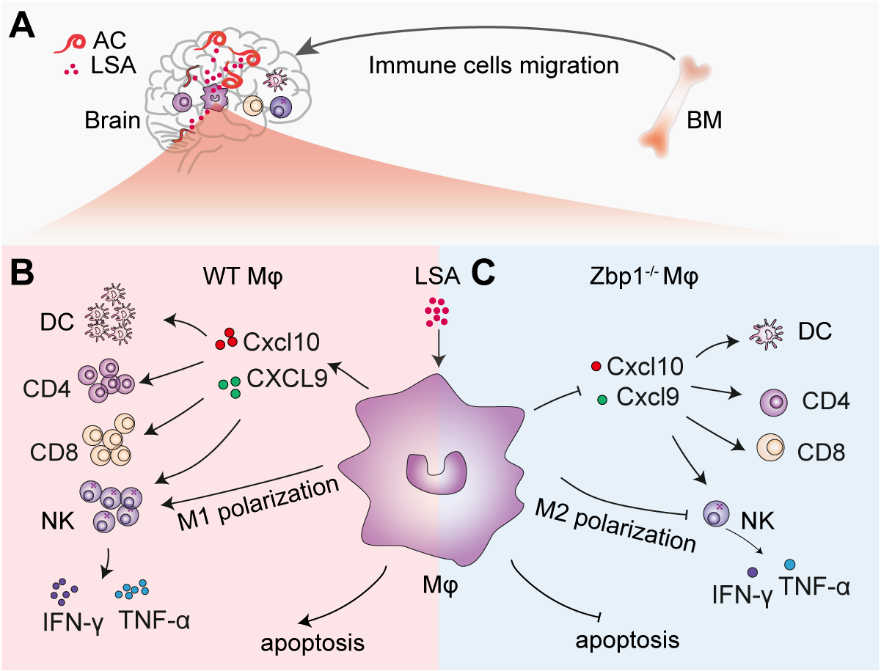


**Figure 9.** Zbp1 Mediates central nervous system inflammation induced by AC via promoting macrophage inflammatory phenotypes**.** (A) Zbp1 enhances AC-induced neuroinflammation by driving the inflammatory polarization of macrophages in the brain. In response to LSA, bone marrow-derived immune cells migrate to the inflamed areas. (B) Under inflammatory stimulation, wild-type macrophages adopt an M1 pro-inflammatory phenotype and release chemokines CXCL10 and CXCL9. This facilitates the infiltration of DCs, CD4⁺ and CD8⁺ T cells, and increases NK cell toxicity, leading to elevated levels of inflammatory cytokines IFN-γ and TNF-α. Additionally, macrophages induce necroptosis via the RIP3/pMlkl pathway, aiding in pathogen clearance. (C) Conversely, Zbp1-deficient macrophages produce less CXCL10 and CXCL9, promoting an M2 anti-inflammatory state. This results in lower levels of IFN-γ and TNF-α and reduced necroptosis, potentially decreasing the inflammatory response. These findings show that Zbp1 controls macrophage polarization and necroptosis, supports lymphocyte infiltration, and increases NK cell toxicity during neuroinflammation.

**Table 1.** RT-qPCR primers used in this study.

| Target sequence | Forward primer sequence | Reverse primer sequence |
| --- | --- | --- |
| Zbp1 | AAGAGTCCCCTGCGATTATTTG | TCTGGATGGCGTTTGAATTGG |
| β-actin | GCTGTCCCTGTATGCCTCT | GTCTTTACGGATGTCAACG |

References

[1] H. Besedovsky, A. del Rey, E. Sorkin, M. Da Prada, R. Burri, C. Honegger, Science (1979) 1983, 221, 564.

[2] J. H. Sampson, M. D. Gunn, P. E. Fecci, D. M. Ashley, Nat Rev Cancer 2020, 20, 12.

[3] M. T. Heneka, M. J. Carson, J. El Khoury, G. E. Landreth, F. Brosseron, D. L. Feinstein, A. H. Jacobs, T. Wyss-Coray, J. Vitorica, R. M. Ransohoff, K. Herrup, S. A. Frautschy, B. Finsen, G. C. Brown, A. Verkhratsky, K. Yamanaka, J. Koistinaho, E. Latz, A. Halle, G. C. Petzold, T. Town, D. Morgan, M. L. Shinohara, V. H. Perry, C. Holmes, N. G. Bazan, D. J. Brooks, S. Hunot, B. Joseph, N. Deigendesch, O. Garaschuk, E. Boddeke, C. A. Dinarello, J. C. Breitner, G. M. Cole, D. T. Golenbock, M. P. Kummer, Lancet Neurol 2015, 14, 388.

[4] D. Giraldo, D. R. Wilcox, R. Longnecker, mBio 2020, 11.

[5] J. Anrather, C. Iadecola, Neurotherapeutics 2016, 13, 661.

[6] A. Venkatesan, B. D. Michael, J. C. Probasco, R. G. Geocadin, T. Solomon, The Lancet 2019, 393, 702.

[7] B. Engelhardt, Results Probl Cell Differ 2006, 43, 259.

[8] J. Kipnis, H. Cohen, M. Cardon, Y. Ziv, M. Schwartz, Proceedings of the National Academy of Sciences 2004, 101, 8180.

[9] L. Adalid-Peralta, B. Sáenz, G. Fragoso, G. Cárdenas, Parasitology 2018, 145, 988.

[10] J. Zhao, Z. Lv, F. Wang, J. Wei, Q. Zhang, S. Li, F. Yang, X. Zeng, X. Wu, Z. Wu, Parasitol Res 2013, 112, 2689.

[11] Z. Li, M. Li, S. X. Shi, N. Yao, X. Cheng, A. Guo, Z. Zhu, X. Zhang, Q. Liu, Journal of Experimental Medicine 2020, 217.

[12] Y. Zhang, Z. Gao, D. Wang, T. Zhang, B. Sun, L. Mu, J. Wang, Y. Liu, Q. Kong, X. Liu, Y. Zhang, H. Zhang, J. He, H. Li, G. Wang, J Neuroinflammation 2014, 11, 79.

[13] Y. Gan, Q. Liu, W. Wu, J.-X. Yin, X.-F. Bai, R. Shen, Y. Wang, J. Chen, A. La Cava, J. Poursine-Laurent, W. Yokoyama, F.-D. Shi, Proc Natl Acad Sci U S A 2014, 111, 2704.

[14] F. Petermann, V. Rothhammer, M. C. Claussen, J. D. Haas, L. R. Blanco, S. Heink, I. Prinz, B. Hemmer, V. K. Kuchroo, M. Oukka, T. Korn, Immunity 2010, 33, 351.

[15] R. Zhang, T. Miao, M. Qin, C. Zhao, W. Wang, C. Zhang, X. Liu, Y. Chen, A. Chen, Y. Wang, Front Cell Infect Microbiol 2021, 11.

[16] Y. Feng, Z. Zhou, Z. Liu, C. Zheng, F. Feng, F. Xie, Z.-D. Wu, Mol Neurobiol 2021, 58, 3968.

[17] L. Wang, S. Zhang, H. Wu, X. Rong, J. Guo, J Leukoc Biol 2019, 106, 345.

[18] M. Casanova-Acebes, E. Dalla, A. M. Leader, J. LeBerichel, J. Nikolic, B. M. Morales, M. Brown, C. Chang, L. Troncoso, S. T. Chen, A. Sastre-Perona, M. D. Park, A. Tabachnikova, M. Dhainaut, P. Hamon, B. Maier, C. M. Sawai, E. Agulló-Pascual, M. Schober, B. D. Brown, B. Reizis, T. Marron, E. Kenigsberg, C. Moussion, P. Benaroch, J. A. Aguirre-Ghiso, M. Merad, Nature 2021, 595, 578.

[19] J. Lan, L. Sun, F. Xu, L. Liu, F. Hu, D. Song, Z. Hou, W. Wu, X. Luo, J. Wang, X. Yuan, J. Hu, G. Wang, Cancer Res 2019, 79, 146.

[20] C. J. Halbrook, C. Pontious, I. Kovalenko, L. Lapienyte, S. Dreyer, H.-J. Lee, G. Thurston, Y. Zhang, J. Lazarus, P. Sajjakulnukit, H. S. Hong, D. M. Kremer, B. S. Nelson, S. Kemp, L. Zhang, D. Chang, A. Biankin, J. Shi, T. L. Frankel, H. C. Crawford, J. P. Morton, M. Pasca di Magliano, C. A. Lyssiotis, Cell Metab 2019, 29, 1390.

[21] H. Guo, R. P. Gilley, A. Fisher, R. Lane, V. J. Landsteiner, K. B. Ragan, C. M. Dovey, J. E. Carette, J. W. Upton, E. S. Mocarski, W. J. Kaiser, Cell Death Dis 2018, 9, 816.

[22] J. Maelfait, L. Liverpool, A. Bridgeman, K. B. Ragan, J. W. Upton, J. Rehwinkel, EMBO J 2017, 36, 2529.

[23] C. L. Pham, N. Shanmugam, M. Strange, A. O’Carroll, J. W. Brown, E. Sierecki, Y. Gambin, M. Steain, M. Sunde, EMBO Rep 2019, 20.

[24] T. H. Pham, K. M. Kwon, Y.-E. Kim, K. K. Kim, J.-H. Ahn, J Virol 2013, 87, 3076.

[25] J. W. Upton, W. J. Kaiser, E. S. Mocarski, Cell Host Microbe 2019, 26, 564.

[26] R. J. Thapa, J. P. Ingram, K. B. Ragan, S. Nogusa, D. F. Boyd, A. A. Benitez, H. Sridharan, R. Kosoff, M. Shubina, V. J. Landsteiner, M. Andrake, P. Vogel, L. J. Sigal, B. R. tenOever, P. G. Thomas, J. W. Upton, S. Balachandran, Cell Host Microbe 2016, 20, 674.

[27] S. Kesavardhana, T. Kuriakose, C. S. Guy, P. Samir, R. K. S. Malireddi, A. Mishra, T.-D. Kanneganti, Journal of Experimental Medicine 2017, 214, 2217.

[28] T. Zhang, C. Yin, D. F. Boyd, G. Quarato, J. P. Ingram, M. Shubina, K. B. Ragan, T. Ishizuka, J. C. Crawford, B. Tummers, D. A. Rodriguez, J. Xue, S. Peri, W. J. Kaiser, C. B. López, Y. Xu, J. W. Upton, P. G. Thomas, D. R. Green, S. Balachandran, Cell 2020, 180, 1115.

[29] K. Newton, K. E. Wickliffe, A. Maltzman, D. L. Dugger, A. Strasser, V. C. Pham, J. R. Lill, M. Roose-Girma, S. Warming, M. Solon, H. Ngu, J. D. Webster, V. M. Dixit, Nature 2016, 540, 129.

[30] Z. Mengying, X. Yiyue, P. Tong, H. Yue, Y. Limpanont, H. Ping, K. Okanurak, W. Yanqi, P. Dekumyoy, Z. Hongli, D. Watthanakulpanich, W. Zhongdao, W. Zhi, L. Zhiyue, Parasit Vectors 2017, 10, 611.

[31] J. L. Guerriero, 2019, pp. 73–93.

[32] S. Yang, M. Zhao, S. Jia, Front Immunol 2023, 14.

[33] F. Askarian, T. Wagner, M. Johannessen, V. Nizet, FEMS Microbiol Rev 2018, 42, 656.

[34] X. Wang, Y. Wang, A. Cao, Q. Luo, D. Chen, W. Zhao, J. Xu, Q. Li, X. Bu, J. Quan, Nat Commun 2023, 14, 6132.

[35] J.-H. Shin, J.-P. Yang, S.-H. Seo, S.-G. Kim, E.-M. Kim, D.-W. Ham, E.-H. Shin, BMB Rep 2020, 53, 478.

[36] N. Kalia, J. Singh, M. Kaur, Immunobiology 2021, 226, 152071.

[37] C. Yan, Q.-Y. Zhou, J. Wu, N. Xu, Y. Du, J. Li, J.-X. Liu, S. Koda, B.-B. Zhang, Q. Yu, H.-M. Yang, X.-Y. Li, B. Zhang, Y.-H. Xu, J.-X. Chen, Z. Wu, X.-Q. Zhu, R.-X. Tang, K.-Y. Zheng, Proceedings of the National Academy of Sciences 2021, 118.

[38] S. B. RASMUSSEN, L. S. REINERT, S. R. PALUDAN, APMIS 2009, 117, 323.

[39] G. Di Benedetto, C. Burgaletto, C. M. Bellanca, A. Munafò, R. Bernardini, G. Cantarella, Cells 2022, 11, 2728.

[40] S. Yang, N. Chang, W. Li, T. Yang, R. Xue, J. Liu, L. Zhang, X. Yao, Y. Chen, H. Wang, L. Yang, J. Huang, L. Li, Cell Death Dis 2023, 14, 175.

[41] L. Liu, H. Guo, A. Song, J. Huang, Y. Zhang, S. Jin, S. Li, L. Zhang, C. Yang, P. Yang, BMC Immunol 2020, 21, 32.

[42] S. Wang, M. Lu, W. Wang, S. Yu, R. Yu, C. Cai, Y. Li, Z. Shi, J. Zou, M. He, W. Xie, D. Yu, H. Jin, H. Li, W. Xiao, C. Fan, F. Wu, Y. Li, S. Liu, Small 2022, 18, e2104112.

[43] J. Luo, J. Wang, J. Zhang, A. Sang, X. Ye, Z. Cheng, X. Li, Cells 2022, 11.

[44] H. Li, T. Zheng, M. Chen, X. Lei, S. Li, X. Chen, S. Wang, Z. Ning, Cell Mol Biol Lett 2024, 29, 83.

[45] N. Somensi, T. K. Rabelo, A. G. Guimarães, L. J. Quintans-Junior, A. A. de Souza Araújo, J. C. F. Moreira, D. P. Gelain, Int Immunopharmacol 2019, 75, 105743.

[46] J. Wei, P. Chen, P. Gupta, M. Ott, D. Zamler, C. Kassab, K. P. Bhat, M. A. Curran, J. F. de Groot, A. B. Heimberger, Neuro Oncol 2019.

[47] C. Marogianni, M. Sokratous, E. Dardiotis, G. M. Hadjigeorgiou, D. Bogdanos, G. Xiromerisiou, Int J Mol Sci 2020, 21, 8421.

[48] J. Neves, P. Sousa‐Victor, FEBS J 2020, 287, 43.

[49] E. Pasciuto, O. T. Burton, C. P. Roca, V. Lagou, W. D. Rajan, T. Theys, R. Mancuso, R. Y. Tito, L. Kouser, Z. Callaerts-Vegh, A. G. de la Fuente, T. Prezzemolo, L. G. Mascali, A. Brajic, C. E. Whyte, L. Yshii, A. Martinez-Muriana, M. Naughton, A. Young, A. Moudra, P. Lemaitre, S. Poovathingal, J. Raes, B. De Strooper, D. C. Fitzgerald, J. Dooley, A. Liston, Cell **2020**, 182, 625.

[50] R. Rua, J. Y. Lee, A. B. Silva, I. S. Swafford, D. Maric, K. R. Johnson, D. B. McGavern, Nat Immunol 2019, 20, 407.

[51] A. De Leo, A. Ugolini, F. Veglia, Cells 2020, 10, 18.

[52] S. Wang, R. Liu, Q. Yu, L. Dong, Y. Bi, G. Liu, Cancer Lett 2019, 452, 14.

[53] T. Hirose, B. W. Scheithauer, M. B. S. Lopes, H. A. Gerber, H. J. Altermatt, M. J. Hukee, S. R. VandenBerg, J. C. Charlesworth, Acta Neuropathol 1995, 90, 387.

[54] C. Zha, X. Meng, L. Li, S. Mi, D. Qian, Z. Li, P. Wu, S. Hu, S. Zhao, J. Cai, Y. Liu, Cancer Biol Med 2020, 17, 154.

[55] A. E. Butterworth, 1985, pp. 143–235.

[56] S. L. Knackstedt, A. Georgiadou, F. Apel, U. Abu-Abed, C. A. Moxon, A. J. Cunnington, B. Raupach, D. Cunningham, J. Langhorne, R. Krüger, V. Barrera, S. P. Harding, A. Berg, S. Patel, K. Otterdal, B. Mordmüller, E. Schwarzer, V. Brinkmann, A. Zychlinsky, B. Amulic, Sci Immunol 2019, 4.

[57] P. H. Gazzinelli-Guimaraes, R. de Queiroz Prado, A. Ricciardi, S. Bonne-Année, J. Sciurba, E. P. Karmele, R. T. Fujiwara, T. B. Nutman, Journal of Clinical Investigation 2019, 129, 3686.

[58] S. Wan, X. Sun, F. Wu, Z. Yu, L. Wang, D. Lin, Z. Li, Z. Wu, X. Sun, J Neuroinflammation 2018, 15, 31.

[59] C. Varol, A. Mildner, S. Jung, Annu Rev Immunol 2015, 33, 643.

[60] T. A. Wynn, K. M. Vannella, Immunity 2016, 44, 450.

[61] P. J. Murray, T. A. Wynn, Nat Rev Immunol 2011, 11, 723.

[62] P. J. Murray, J. E. Allen, S. K. Biswas, E. A. Fisher, D. W. Gilroy, S. Goerdt, S. Gordon, J. A. Hamilton, L. B. Ivashkiv, T. Lawrence, M. Locati, A. Mantovani, F. O. Martinez, J.-L. Mege, D. M. Mosser, G. Natoli, J. P. Saeij, J. L. Schultze, K. A. Shirey, A. Sica, J. Suttles, I. Udalova, J. A. van Ginderachter, S. N. Vogel, T. A. Wynn, Immunity 2014, 41, 14.

[63] T. Kuriakose, T.-D. Kanneganti, Trends Immunol 2018, 39, 123.

[64] M. Zheng, T. Kanneganti, Immunol Rev 2020, 297, 26.

[65] M. Momota, P. Lelliott, A. Kubo, T. Kusakabe, K. Kobiyama, E. Kuroda, Y. Imai, S. Akira, C. Coban, K. J. Ishii, Int Immunol 2020, 32, 203.

[66] K. J. Pittman, P. W. Cervantes, L. J. Knoll, Infect Immun 2016, 84, 3063.

[67] X. Du, W. Ge, R. Jing, L. Pan, Int Immunopharmacol 2019, 77, 105944.

[68] K. De Vlaminck, H. Van Hove, D. Kancheva, I. Scheyltjens, A. R. Pombo Antunes, J. Bastos, M. Vara-Perez, L. Ali, M. Mampay, L. Deneyer, J. F. Miranda, R. Cai, L. Bouwens, D. De Bundel, G. Caljon, B. Stijlemans, A. Massie, J. A. Van Ginderachter, R. E. Vandenbroucke, K. Movahedi, Immunity 2022, 55, 2085.

[69] J. R. Plemel, J. A. Stratton, N. J. Michaels, K. S. Rawji, E. Zhang, S. Sinha, C. S. Baaklini, Y. Dong, M. Ho, K. Thorburn, T. N. Friedman, S. Jawad, C. Silva, A. V. Caprariello, V. Hoghooghi, J. Yue, A. Jaffer, K. Lee, B. J. Kerr, R. Midha, P. K. Stys, J. Biernaskie, V. W. Yong, Sci Adv 2020, 6.

[70] T. Zhang, C. Yin, D. F. Boyd, G. Quarato, J. P. Ingram, M. Shubina, K. B. Ragan, T. Ishizuka, J. C. Crawford, B. Tummers, D. A. Rodriguez, J. Xue, S. Peri, W. J. Kaiser, C. B. López, Y. Xu, J. W. Upton, P. G. Thomas, D. R. Green, S. Balachandran, *Cell* 2020, *180*, 1115.

[71] J. Du, Y. Liu, G. Lan, Y. Zhou, Y. Ni, K. Liao, F. Zheng, Q. Cheng, G. Shi, X. Su, *Cell Death Dis* 2023, *14*, 432.

[72] X.-Y. Chen, Y.-H. Dai, X.-X. Wan, X.-M. Hu, W.-J. Zhao, X.-X. Ban, H. Wan, K. Huang, Q. Zhang, K. Xiong, *Molecules* 2022, *28*.

[74] H. England, H. R. Summersgill, M. E. Edye, N. J. Rothwell, D. Brough, *J Biol Chem* 2014, *289*, 15942.

[74] C. Peng, G. Tu, J. Wang, Y. Wang, P. Wu, L. Yu, Z. Li, X. Yu, *Cell Death Dis* 2023, *14*, 155.

[75] A. Nease, E. Malovic, N. Kondru, H. Jin, V. Anantharam, A. Kanthasamy, A. Kanthasamy, *The FASEB Journal* 2019, *33*.

[76] H. Guo, R. Chen, P. Li, Q. Yang, Y. He, *Mol Cell Biochem* 2023, *478*, 2849.

[77] S. Nagata, Annu Rev Immunol 2018, 36, 489.

[78] M. F. Linton, V. R. Babaev, J. Huang, E. F. Linton, H. Tao, P. G. Yancey, Circulation Journal 2016, 80, 2259.

[79] P. Qiu, Y. Liu, K. Chen, Y. Dong, S. Liu, J. Zhang, Ann Transl Med 2021, 9, 974.

[80] J. Lee, M. Hartman, H. Kornfeld, Yonsei Med J 2009, 50, 1.

[81] A. Polykratis, A. Martens, R. O. Eren, Y. Shirasaki, M. Yamagishi, Y. Yamaguchi, S. Uemura, M. Miura, B. Holzmann, G. Kollias, M. Armaka, G. van Loo, M. Pasparakis, Nat Cell Biol 2019, 21, 731.

[82] P. Orning, D. Weng, K. Starheim, D. Ratner, Z. Best, B. Lee, A. Brooks, S. Xia, H. Wu, M. A. Kelliher, S. B. Berger, P. J. Gough, J. Bertin, M. M. Proulx, J. D. Goguen, N. Kayagaki, K. A. Fitzgerald, E. Lien, Science 2018, 362, 1064.

[83] M. D. Stutz, C. C. Allison, S. Ojaimi, S. P. Preston, M. Doerflinger, P. Arandjelovic, L. Whitehead, S. M. Bader, D. Batey, M.-L. Asselin-Labat, M. J. Herold, A. Strasser, N. P. West, M. Pellegrini, Immunity 2021, 54, 1758.

[84] X. Zhang, D. He, S. Gao, Y. Wei, L. Wang, Mol Med Rep 2019.

[85] S. Y. Nuñez, A. Ziblat, F. Secchiari, N. I. Torres, J. M. Sierra, X. L. Raffo Iraolagoitia, R. E. Araya, C. I. Domaica, M. B. Fuertes, N. W. Zwirner, The Journal of Immunology 2018, 200, 1008.

[86] S. Viel, A. Marçais, F. S.-F. Guimaraes, R. Loftus, J. Rabilloud, M. Grau, S. Degouve, S. Djebali, A. Sanlaville, E. Charrier, J. Bienvenu, J. C. Marie, C. Caux, J. Marvel, L. Town, N. D. Huntington, L. Bartholin, D. Finlay, M. J. Smyth, T. Walzer, Sci Signal 2016, 9.

[87] X.-Q. Wang, W.-J. Zhou, X.-X. Hou, Q. Fu, D.-J. Li, Cell Mol Immunol 2018, 15, 1038.

[88] H. Zhou, Y. Lu, H. Wei, Y. Chen, Y. Limpanon, P. Dekumyoy, P. Huang, P. Shi, Z. Lv, PLoS Negl Trop Dis 2022, 16, e0010461.

Supplementary material


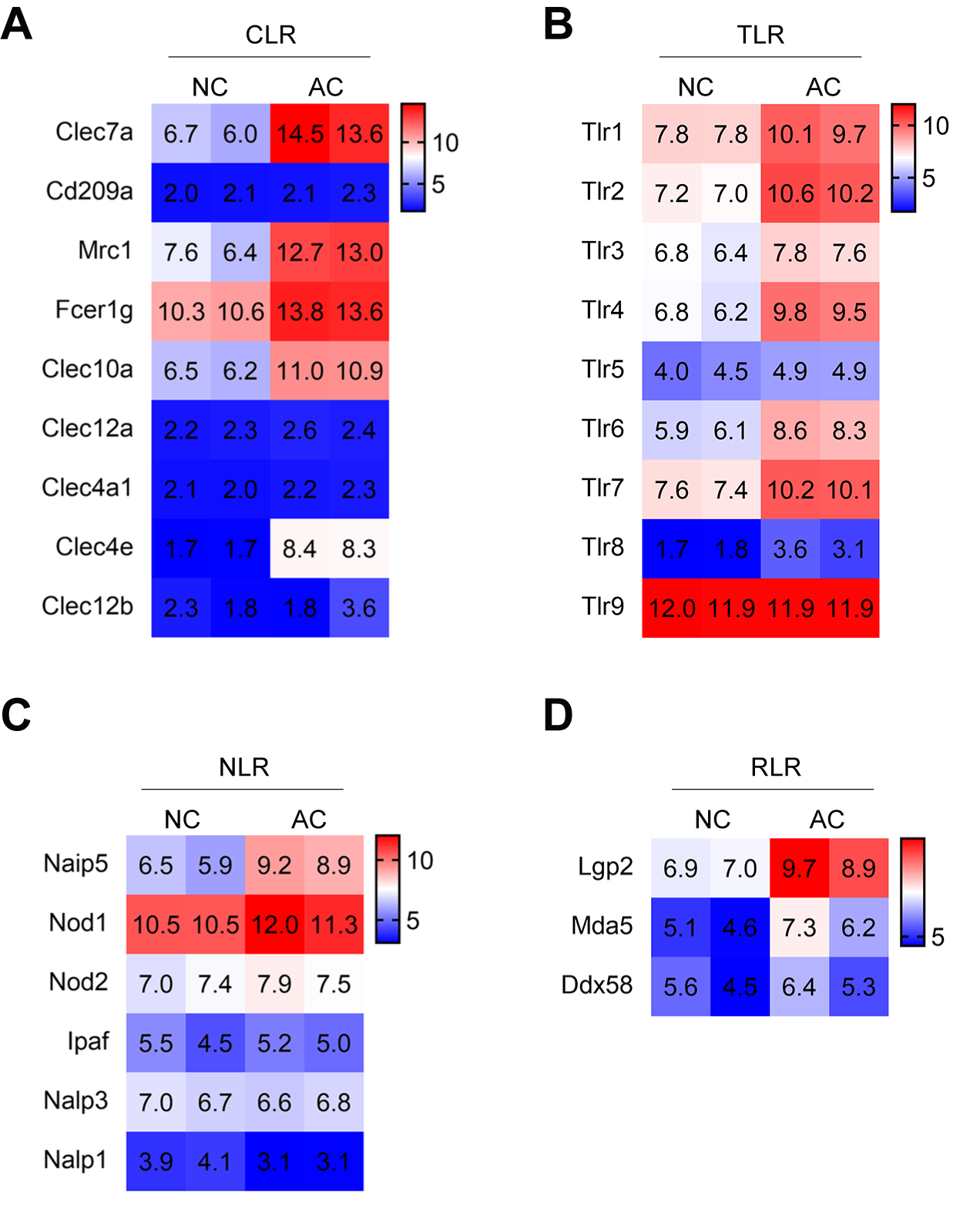


**Figure S1.** The expression profile of PRR in C57BL/6 mouse brain after AC infection. (A-E) The expression profiles of CLR(A), TLR(B), NLR(C) and RLR(D) in the brain of infected mouse displayed by heatmap analysis.


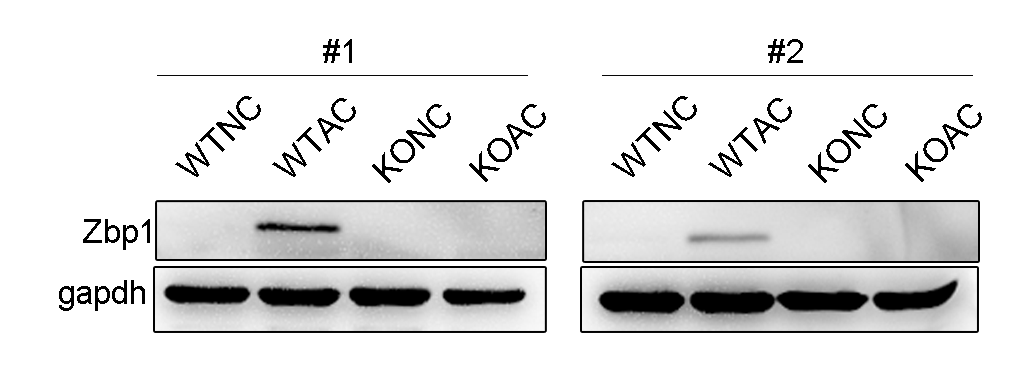


**Figure S2.** Validation of Zbp1 knock-out mice with brain tissue upon AC infection.


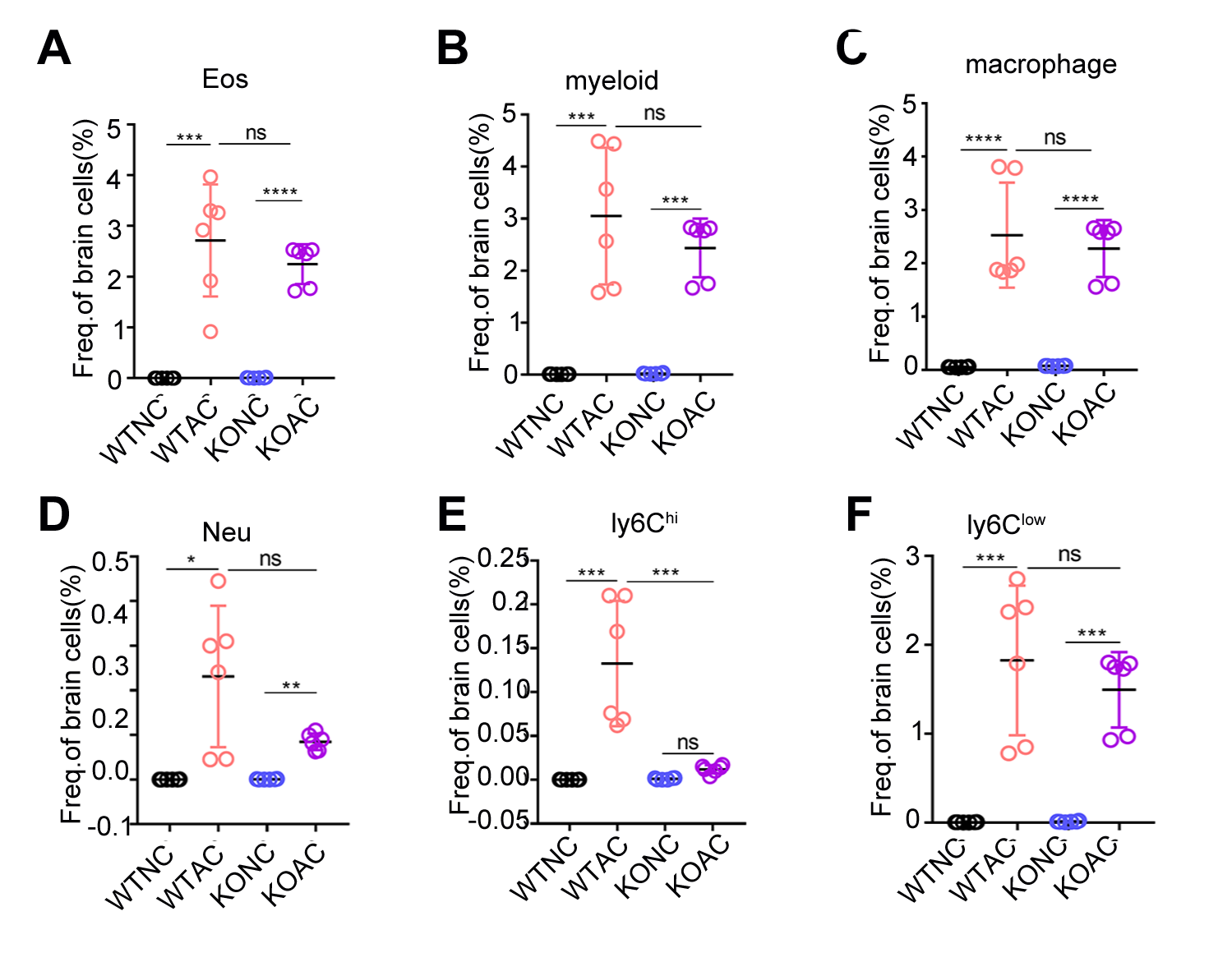


**Figure S3.** The proportion of Eos (A) Myeloid cells. (B) Macrophage. (C) Neu. (D-E) Ly6C^hi^ and ly6C^low^. (F) Monocytes in mouse brain after AC infection was measured by flow cytometric analysis. n=5-6 per group. *** P < 0.001, **** P < 0.0001.


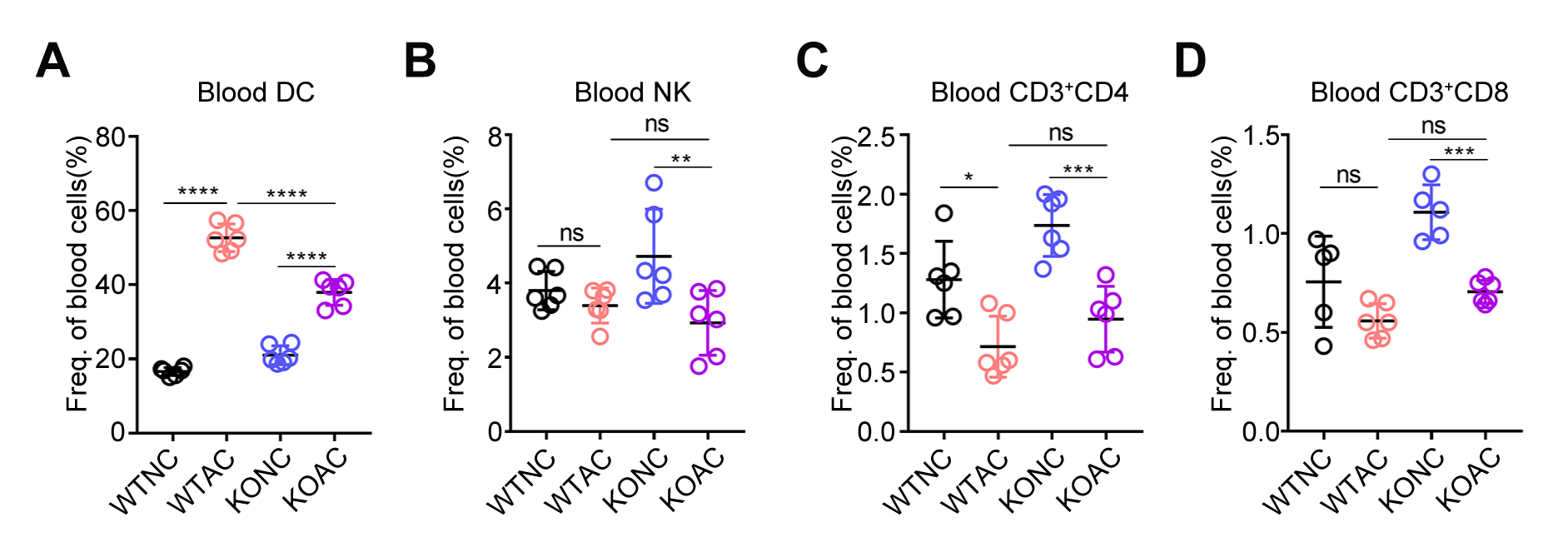


**Figure S4.** The proportion of DCs(B), NK (C), CD3^+^CD4(D) and CD3^+^CD8 T (E) cells in mouse peripheral blood from different groups after AC infection. n=5-6 per group. * P < 0.05，** P < 0.01，*** P < 0.001，**** P < 0.0001.
